# Anti-Inflammatory Derivatives with Dual Mechanism of Action from the Metabolomic Screening of *Poincianella pluviosa*

**DOI:** 10.3390/molecules24234375

**Published:** 2019-11-29

**Authors:** Olívia da S. Domingos, Bianca G. V. Alcântara, Mário F. C. Santos, Tatiane C. S. Maiolini, Danielle F. Dias, João L. Baldim, João Henrique G. Lago, Marisi G. Soares, Daniela A. Chagas-Paula

**Affiliations:** 1 Instituto de Química–Universidade Federal de Alfenas, Alfenas 37130-001, MG, Brazil; oliviadomingos@hotmail.com (O.d.S.D.); bianca.vasconcelos.alcantara@gmail.com (B.G.V.A.); mariosantos408@gmail.com (M.F.C.S.); tatychryss@gmail.com (T.C.S.M.); danielle.dias@unifal-mg.edu.br (D.F.D.); jotaelebaldim@gmail.com (J.L.B.); 2 Instituto Federal de de Educação, Ciência e Tecnologia do Sul de Minas Gerais-IFSULDEMINAS, Muzambinho 37890-000, MG, Brazil; 3 Centro de Ciências Naturais e Humanas, Universidade Federal do ABC, Santo Andre 09606-045, SP, Brazil or

**Keywords:** *Poincianella pluviosa*, anti-inflammatory, flavonoids, metabolomics

## Abstract

Metabolomics approaches have become fundamental strategies for the analysis of complex mixtures, guiding the isolation of target compounds by focusing on unpublished or promising pharmacological properties. The discovery of novel anti-inflammatory agents is important due to several limitations regarding their potency, efficacy, and adverse effects. Thus, novel anti-inflammatory candidates are essential, aiming to find agents with better mechanisms of action. In this context, extracts from *Poincianella pluviosa* var. *peltophoroides* demonstrated significant in vivo anti-inflammatory potential. Thus, metabolomics analysis based on UHPLC-UV-HRFTMS data was performed for the identification of biomarkers with anti-inflammatory properties. Metabolomics-guided chromatographic process led to the isolation of novel compounds 4‴-methoxycaesalpinioflavone and 7-methoxycaesalpinioflavone, as well as known derivatives rhuschalcone VI and caesalpinioflavone. Isolated compounds caused edema inhibition and neutrophil recruitment. Two of them showed better efficacy than reference drugs (indomethacin and dexamethasone). Results of in vivo experiments corroborated those obtained through metabolomics and statistical analyses guiding the isolation of substances of interest.

## 1. Introduction

Natural products are of great relevance for the discovery and development of new drugs due to the variety of secondary metabolites with biological importance which are biosynthesized by living organisms [1,2]. Metabolomics, and other so-called omics sciences, such as genomics, transcriptomics, and proteomics, analyzes a biological system using the complete set of metabolites in a biological system. These strategies make it possible to detect important variables that possibly affect biological processes or activities based on metabolite-centered data [3]. Metabolomics approaches allow the investigation of highly complex biological matrices using modern analytical methods. The interpretation and mining of the large amounts of spectroscopic and/or chromatographic data generated by these approaches requires the use of multivariate analysis for sample classification, monitoring changes in metabolite composition [4], or to determine compounds associated with pharmacological properties [5,6].

Anti-inflammatory drugs are some of the most widely-used therapeutic agents in the world, since inflammation is related to several diseases resulting in pronounced morbidity. For the treatment of acute and chronic inflammation, classic nonsteroidal anti-inflammatory drugs (NSAIDs) such as aspirin, ibuprofen, and indomethacin are widely used, which act by inhibiting the isoforms of cyclooxygenase, COX-1, and COX-2. However, due to the adverse effects of COX-1 inhibition by these drugs, such as renal and gastrointestinal toxicity, selective COX-2 inhibitors have been developed [7]. Among NSAIDs, piroxicam, meloxicam, naproxen, and nimesulide are first-generation selective COX-2 inhibitors, while celecoxib, rofecoxib, etoricoxib, valdecoxib, parecoxib, and lumiracoxib are highly selective second-generation alternatives [7,8,9]. Nevertheless, there are adverse cardiovascular effects resulting from the use of these drugs [7].

There are also steroidal anti-inflammatory drugs (SAIDs) or corticosteroids, such as prednisone, dexamethasone, and betamethasone, which exhibit powerful anti-inflammatory and immunosuppressive effects, but when used in long-term treatment, they present serious side effects including osteoporosis, cataracts, diabetes, bone fractures, hypertension, peripheral resistance to insulin, hyperglycemia, growth retardation in children, decreased libido, impotence, gastric irritation, peptic ulcer, glaucoma, sleep disorders, irritability, and depression, among others [10,11].

Mostly, anti-inflammatory treatments target only the arachidonic acid (AA) cascade, and are accompanied by many side effects. Thus, it is necessary to find other strategies for multi-target drug development, aiming to find satisfactory treatments of inflammation while minimizing adverse effects [12,13]. In this scenario, agents that can inhibit both COX and 5-LOX pathways have the potential to display higher anti-inflammatory activities with fewer adverse effects [7]. Both enzymes can metabolize AA during inflammation; whereas COX is inhibited, 5-LOX will still produce leukotrienes, which are responsible for neutrophil recruitment. This is associated with gastric side effects, the main side effect of COX-1 inhibitors [7]. The inhibition of prostaglandin and leukotriene biosynthesis by COX and 5-LOX, respectively, can be evaluated by the in vivo anti-inflammatory assay of croton oil-induced ear edema [14]. The inhibition of COX pathway is observed by reducing edema in ears of animals treated with anti-inflammatory agents. Concomitantly, a decreasing in the activity of myeloperoxidase (MPO, an enzyme expressed by neutrophils) indicates that fewer neutrophils are recruited, suggesting the inhibition of 5-LOX pathway [14,15,16].

The species *Poincianella pluviosa* (Leguminosae) demonstrated significant anti-inflammatory potential [17,18], besides other important pharmacological properties reported [18,19,20,21,22,23,24,25]. Thus, *P. pluviosa* is a promising species in the investigation of new anti-inflammatory agents using metabolomics approaches and in vivo models which are able to evaluate its important mechanisms of action.

## 2. Results and Discussion

### 2.1. Anti-Inflammatory Activity Evaluation Analysis

**Figure 1:**
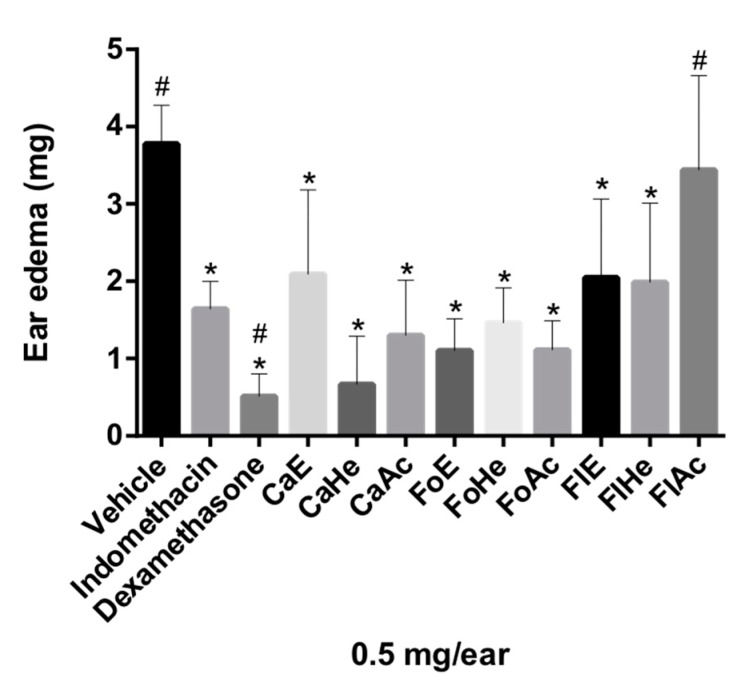
Evaluation of anti-inflammatory activity of fractions of *P. pluviosa* in the ear edema model in mice. The results were analyzed by one-way ANOVA, followed by Dunett’s multiple comparison test. * *p* ≤ 0.05 compared to vehicle and # *p* ≤ 0.05 compared to indomethacin.

The anti-inflammatory activity of the hexane, EtOAc, and the hydroethanolic fractions of the flowers (FlHe, FlAc, and FlE, respectively), leaves (FoHe, FoAc, and FoE), and stem bark (CaHe, CaAc and CaE) of *P. pluviosa* were evaluated by the croton oil-induced ear edema assay. Croton oil is an irritant, responsible for causing cellular damage and activating phospholipase A2, which causes AA release, the precursor of inflammatory mediator prostaglandins and leukotrienes. These mediators are involved in the formation of edema and leukocyte migration, respectively [26]. After the application of croton oil to the left ear of mice, it was possible to observe an apparent inflammatory response, due to the formation of edema and flushing, i.e., the observable cardinal signs of inflammation [26]. All tested fractions, except FlAc, presented significant ear edema reduction (Figure 1). Fractions and indomethacin were considered statistically similar in Dunnett’s multiple comparison tests (*p* ≤ 0.05), suggesting that stem bark, leaves, and flowers of *P. pluviosa* could all contain anti-inflammatory compounds.

In the ear edema experimental model, anti-inflammatory activity can also be verified through the quantification of MPO, which is a marker in inflamed tissues. MPO is an enzyme found in neutrophils [27,28]. Thus, a decrease in MPO in the ear indicates that fewer neutrophils were recruited, so the 5-LOX pathway can be inhibited [6,14,16]. MPO activity was quantified by the absorbance values obtained in the test, which are proportional to the enzyme concentration in samples.

**Figure 2:**
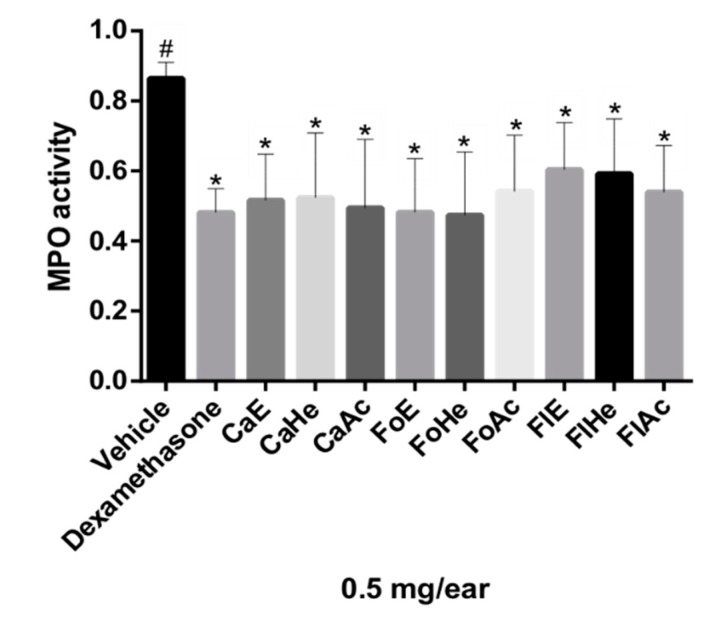
Effect of fractions of *P. pluviosa* on neutrophil recruitment measured via myeloperoxidase quantification (MPO). The results were analyzed by one-way ANOVA, followed by a Dunett’s multiple comparison test; results are expressed as mean ± SEM. * *p* ≤ 0.05 compared to vehicle and # *p* ≤ 0.05 compared to dexamethasone.

Neutrophils are circulating polymorphonuclear leukocytes; their main functions are phagocytosis and the elimination of pathogens. Treatment with glucocorticoids, such as dexamethasone, can suppress neutrophil migration during the inflammatory response by reducing the expression of adhesion molecules [29]. All fractions tested significantly inhibited MPO compared to dexamethasone (statistically similar), as shown with a Dunnett’s multiple comparison test (*p* ≤ 0.05) (Figure 2).

Therefore, all fractions of *P. pluviosa* have shown to inhibit inflammatory processes which include the COX and 5-LOX pathways, with exception to FlAc (which did not lead to a reduction of ear edema).

The chromatographic method used in these metabolomics studies yielded a good distribution of the constituents from all fractions through the chromatogram. This is a very comprehensive method that has been used successfully for the analysis of fractions of species from other families [6]. Chromatographic data from each fraction were treated in MZmine 3.0 and exported as .csv format file for multivariate analysis in the software SIMCA-P in order to identify possible biomarkers to the anti-inflammatory activity. All statistical analyses were performed with the chromatographic data of mass detection in the positive and negative modes separately. For simplification, the results referring to the negative mode will be presented, where 536 substances were detected, while in positive mode, only 52 substances were detected. Thus, it can be inferred that most of the metabolites present in the evaluated fractions contains acid groups, which explains their ionization mainly occurring by the loss of protons detected by the experiments in negative mode.

The retention time and *m*/*z* are called independent x variables or predictor variables; anti-inflammatory activity is the dependent variable associated with the y axis. Thus, the statistical analysis employed in this study aimed to verify the relationship between detected metabolites (x variables) and pharmacological properties (y variables) exhibited by the fractions. Initially, a Principal Component Analysis (PCA, unsupervised statistical analysis) was performed, indicating that samples were organized according to their similarity degrees based on their chemical compositions (Supplementary Materials) and pharmacological activity. The method was well fitted with R2 value > 0.5 (R2X = 0.53) [30,31]. On the PCA score plot (Supplementary Information), the sample FlAc, the only sample that showed inhibition of neutrophil recruitment (NR) but did not inhibit the ear edema, was projected outside the hotteling’s T^2^ ellipse. It suggests that FlAc did not contain potential compounds for dual inhibition of both NR and edema. This analysis also indicated that the leaf fractions (FoE, FoAc, FoHe) and stem fractions (CaE, CaAc, CaHe) contain compounds which are able to present dual inhibition of edema and NR, and should be used in the isolation of anti-inflammatory compounds.

**Figure 3:**
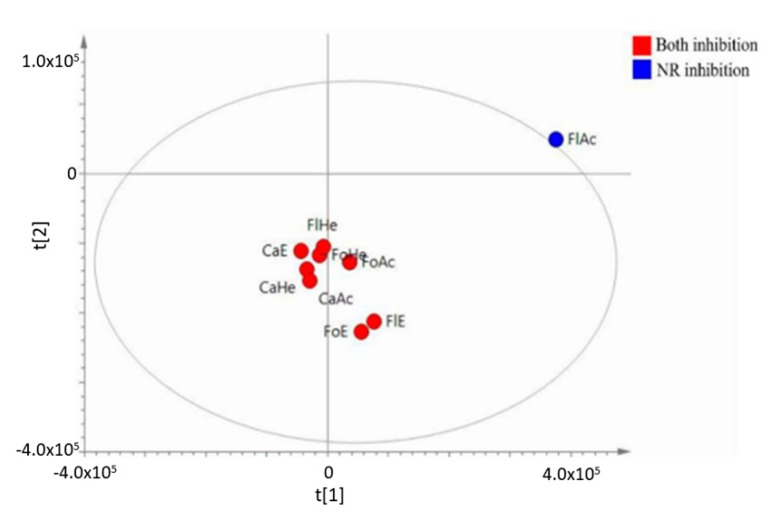
Distribution of fractions of *P. pluviosa* according to anti-inflammatory activity obtained in PLS-DA analysis (R2X = 0.617, R2Y = 0.993, and Q2 = 0.752). Both inhibition: edema and neutrophil recruitment inhibition; NR inhibition: neutrophil recruitment inhibition.

Thus, a Partial Least Square-Discriminant Analysis (PLS-DA) method was created in the SIMCA-P software, reaching R2X = 0.617, R2Y = 0.993, and Q2 = 0.752, to detect possible biomarkers (Figure 3). The classes of each sample, which, in this case, are their respective pharmacological properties, were previously informed for building the model. Fractions CaE, CaAc, CaHe, FoE, FoAc, FoHe, FlE, and FlHe, which presented inhibition of both ear edema and neutrophil recruitment, were defined as both inhibitions, whereas the fraction FlAc, which only showed inhibition of neutrophil recruitment, was defined as NR inhibition. A similar approach was carried out with Orthogonal Partial Least Square (OPLS). However, this method would violate the principle of parsimony [32], since similar values of R2X, R2Y, and Q2 were found to those of the simpler model, i.e., the PLS-DA. The cross-validation value obtained for PLS-DA indicated that the method was robust: Q2 > 0.5 [30,31] (Q2 = 0.752).

The results of the PLS-DA model indicated variables which are important for the projection (VIPs). Thus, it was possible to identify variables with strong influence on the model projection. In this context, the most important metabolites are those with values of VIP > 1, which could be promising biomarkers for the anti-inflammatory activity [30,31]. Additionally, coefficient values allowed us to examine of the role of each variable for activity, where positive coefficient values were associated with a positive influence exerted by the variable against the inflammatory process [32,33]. 

The important variables for anti-inflammatory activity through the inhibition of edema and neutrophil recruitment were predominantly phenolic compounds, as expected for species of the genus *Poincianella* [17,22,34,35]. All VIPs were registered (Table 1) and associated mostly with major compounds. They were dereplicated using the comprehensive DNP database and specific in-house database of the *Poincianella* genus. It was observed that some VIPs had several hits already identified in the species of other genera. However, many VIPs didn’t present hits, even employing comprehensive databases, suggesting that these are possibly novel substances (Table 1). In this way, chromatographic procedures were performed to provide the isolation of these new substances, which are of pharmacological interest.

**Figure 4:**
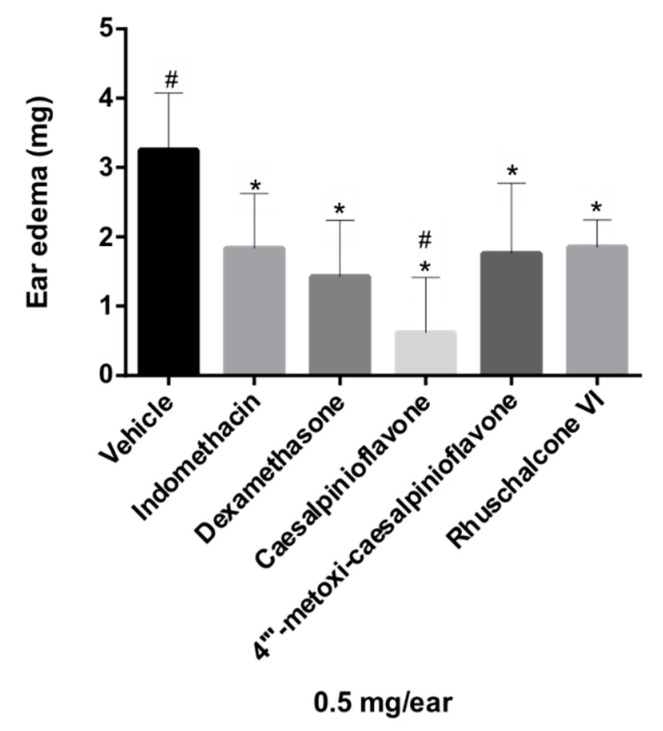
Evaluation of the anti-inflammatory activity of compounds **1–4** in the ear edema model in mice. Results were analyzed by one-way ANOVA, followed by Dunett’s multiple comparison test. * indicates levels of significance compared to the vehicle (*p* ≤ 0.05); # indicates levels of significance compared to the reference drug, indomethacin (*p* ≤ 0.05).

**Figure 5:**
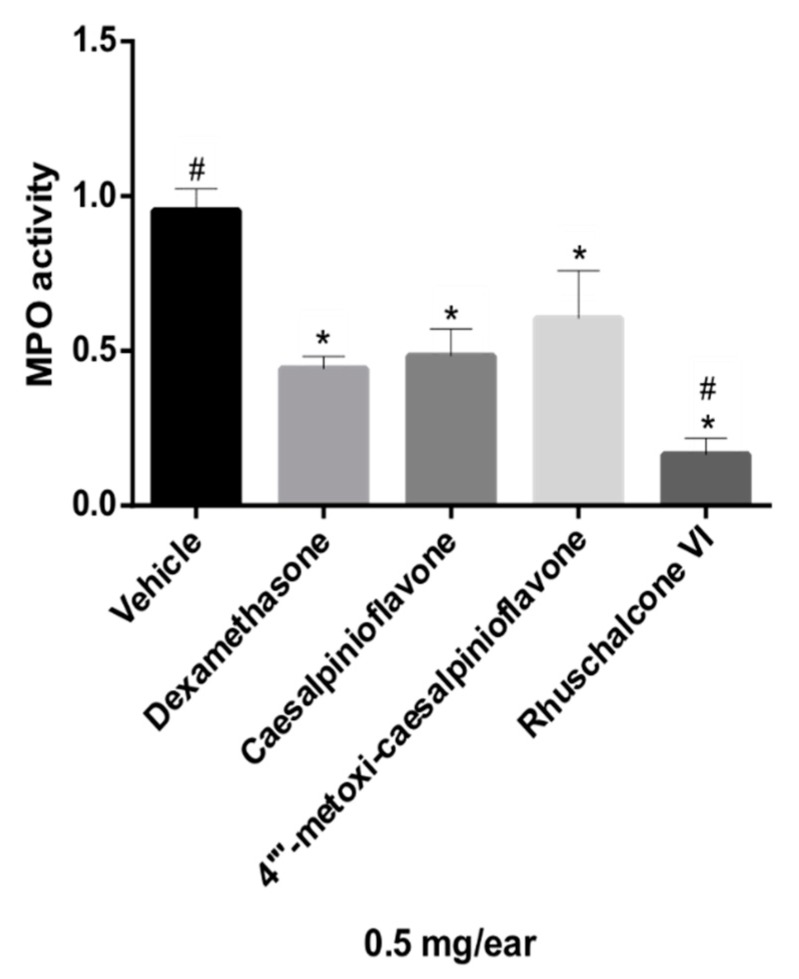
Effect of compounds **1–4** on neutrophil recruitment measured via myeloperoxidase quantification (MPO). Results were analyzed by one-way ANOVA, followed by Dunett’s multiple comparison test and expressed as mean ± SEM. * *p* ≤ 0.05 compared to vehicle and # *p* ≤ 0.05 compared to dexamethasone.

The biflavonoid caesalpinioflavone (**1**), with *m*/*z* equal to 525.1186 (Table 1), previously described in *P. pluviosa* [24], was dereplicated as an important variable for the projection of the anti-inflammatory activity model (VIP = 1.4, Table 1). This compound was positively correlated with dual anti-inflammatory activity, with correlation coefficient = +5.9 × 10^7^. Caesalpinioflavone is a major metabolite of the ethyl acetate and hydroethanolic fractions of the stem bark of the species, which showed significant anti-inflammatory potential. Caesalpinioflavone was evaluated for the first time in an ear edema model; MPO and experimental results confirm its anti-inflammatory potential, as suggested by the statistical model (Figure 4 and Figure 5).

Only one hit was found for the variables that represent *m*/*z* 539.1342 and R_t_ 19.9 min (ID = 6) and 19.1 min (ID = 100) with VIP value of 1.3 and 1.5 (Table 1), respectively, and positive correlation with both inhibition group. The hit was described in the species of another family, and it is unusual in the species of Leguminosae, which suggests that the metabolites could be unknown. Thus, two isomers were isolated and identified as novel methoxyl derivatives of caesalpinioflavone (compounds **2** and **3**). The correlation coefficients found for compounds **2** and **3** were +5.50 × 10^7^ and +4.70 × 10^7^, respectively.

The variable with *m*/*z* 509.1242 (VIP = 0.9), even though it falls in a gray area to be defined as being important in the separation of the groups on the PLS-DA model, could also be important (Table 1), since it shows a strong positive correlation with anti-inflammatory activity, with correlation coefficient = +2.90 × 10^7^. Compound **4** was present in large quantities in the evaluated fraction, so it was also isolated and subjected to anti-inflammatory evaluations.

Compounds **1**, **2**, and **4** had their anti-inflammatory activity confirmed for the inhibition of both edema and neutrophil recruitment (Figure 4 and Figure 5), corroborating the relevance of metabolomics strategies to guide the isolation of new metabolites of pharmacological relevance. There were reductions of 69.23%, 47.38%, and 43.07% of ear edema in the groups treated with compounds **1**, **2**, and **4**, respectively, i.e., the values were significantly different from the vehicle (Figure 4) compared to that presented by reference drugs (indomethacin and dexamethasone, 43.38% and 56.00%, respectively). However, biomarker **1** showed higher anti-edematogenic properties than indomethacin. Compounds **1**, **2**, and **4** also presented significant results for the inhibition of neutrophil recruitment while compared to the vehicle. Biomarker **4** displayed higher inhibition of neutrophil recruitment than dexamethasone, a potent SAID.

Thus, metabolomics tools clearly directed the isolation of substances of pharmacological interest, optimizing phytochemical procedures for the isolation of new derivatives of caesalpinioflavone. In addition, their anti-inflammatory mechanisms were better than those of NSAIDs and SAIDs, which could be useful as lead compounds and for the development of new anti-inflammatory agents with higher efficacy and fewer side effects. These derivatives were shown to be active when investigated individually (Figure 4 and Figure 5); a synergic or additive effect may possibly occur on the active extract and fractions *P. pluviosa* (Figure 1 and Figure 2).

### 2.2. Chemical Characterization of Compounds ***1**–**4***

**Figure 6:**
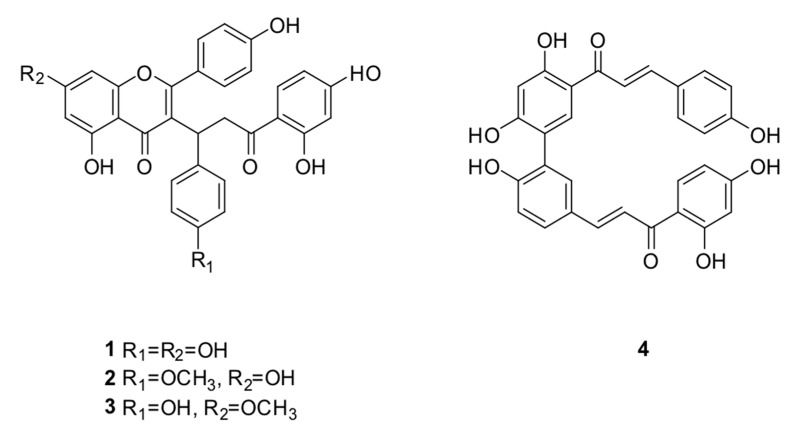
Compounds **1–4** isolated from *P. pluviosa.*

Compound **2** (Figure 6) was obtained as an amorphous yellow solid and the HRESIMS spectrum (Supplementary Materials) exhibited [M − H]^−^ at *m*/*z* 539.1346 (calcd. for C_31_H_23_O_9_, 539.1342). Its molecular formula accounted for 20 unsaturation degrees. The ^1^H NMR spectrum (Supplementary Materials) exhibited proton systems which were characteristic for the two AA’BB’ spin system of eight aromatic protons (ring B and A’) at δ_H_ 6.73 (2H, d, *J* = 8.6 Hz), 6.98 (2H, d, *J* = 8.6 Hz), 6.81 (2H, d, *J* = 8.7 Hz), and 7.08 (2H, d, *J* = 8.7 Hz)], an ABX spin system of three aromatic protons (ring B’) at δ_H_ 6.17 (1H, d, *J* = 2.2 Hz), 6.15 (1H, dd, *J* = 8.6, 2.2 Hz) and 7.18 (1H, d, *J* = 8.6 Hz) as well as a tetrasubstituted phenyl group with a pair of meta-coupled protons (ring A) at δ_H_ 6.26 (1H, d, *J* = 2.0 Hz) and 6.17 (1H, d, *J* = 2.0 Hz)]. The singlets at δ_H_ 3.66, 12.66, and 11.87 are indicative of methoxyl and hydroxyl groups, while signs at δ_H_ 2.97 (1H, dd, *J* = 13.9, 6.7 Hz), 3.44 (1H, m), and 4.57 (1H, t, *J* = 6.7 Hz) were assigned to hydrogens of methylene and methine carbons, respectively.

**Figure 7:**
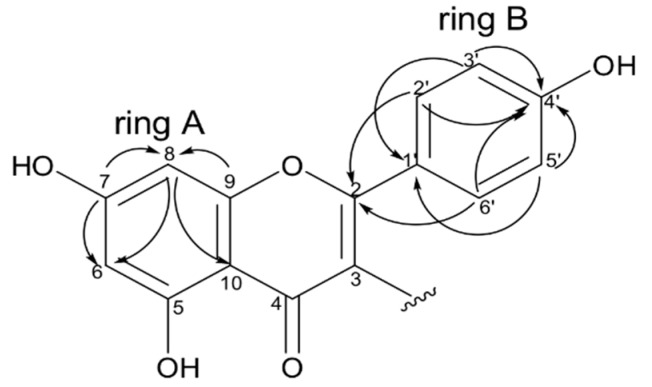
Key HMBC correlations observed for flavone moiety (substructures I).

The ^13^C NMR spectrum (Supplementary Materials) revealed the presence of 28 sp^2^ hybridized carbons, including two carbonyls at δ_C_ 181.1 (C-4) and 201.3 (C-4″), thirteen quaternary carbons, of which eight were substituted by an oxygen atom at δ_c_ 157.7, 158.1, 160.0, 161.9, 163.5, 164.4 (2C), and 165.2, and others by a carbon atom at δ_C_ 99.4, 113.1, 119.4, 122.8, and 132.2, as well as 13 methine carbons at δ_C_ 94.1, 103.5, 103.0, 131.7, 108.2, 114.0 (2C), 115.7 (2C), 130.7 (2C), and 130.4 (2C). Moreover, signal of methoxyl carbon was observed at δ_C_ 55.4 as well as peaks attributed to methylene and methine carbons were detected at δ_C_ 34.5 and 48.4 respectively. A detailed analysis of the HMBC data led to the definition of two substructures, i.e., I and II. Substructure I (Figure 7) showed HMBC correlations of hydrogens H-2′/H-6′ (δ_H_ 7.08, ring B) to the oxygenated quaternary carbon at δ_C_ 164.4 (C-2), of hydrogens H-3′/H-5′ (δ_H_ 6.81, ring B) with the carbon at δC 160.0 (C-4′). Moreover, correlations were observed between the hydrogens and carbons inside ring A, as well as ring B. Theses correlations suggest the presence of a flavone moiety in the structure of the molecule.

**Figure 8:**
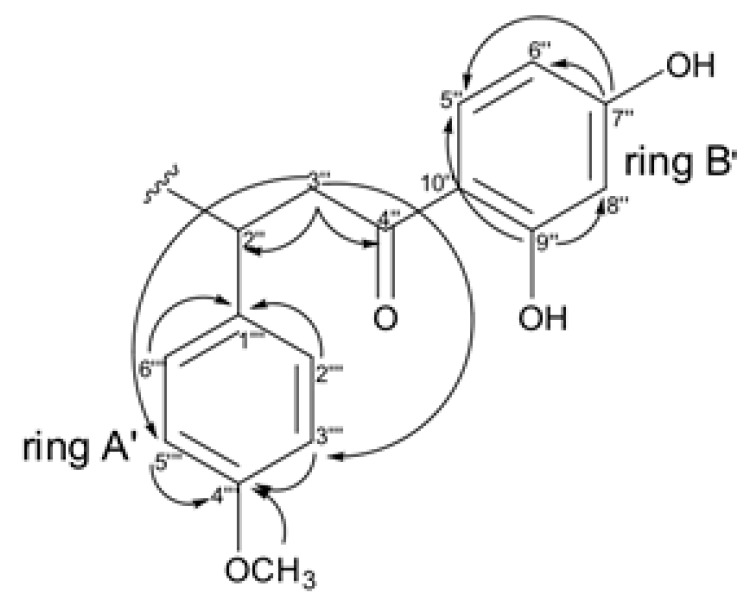
Key HMBC correlations observed for chalcone moiety (substructures II).

Similarly, the correlations observed on HMBC for ring A’ end B’ suggest the presence of the chalcone moiety (Figure 8), where the methoxyl group was positioned through of correlation of δ_H_ 3.66 (CH_3_O) with the carbon at C-4‴ (δ_C_ 158.1) on the ring A’.

**Figure 9:**
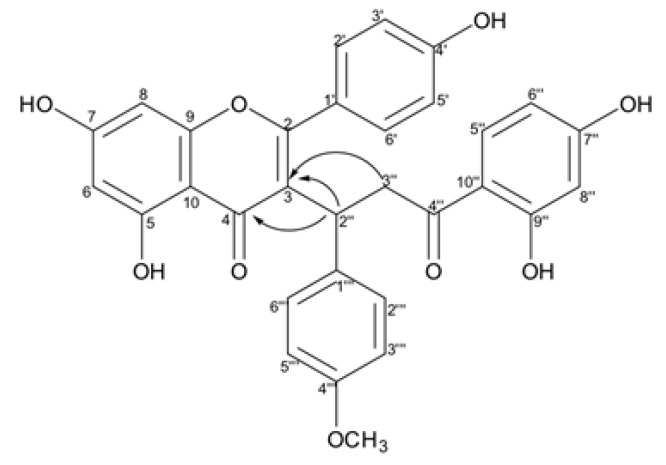
Key HMBC cross-peaks observed for the connection between chalcone e flavone moieties.

The connection between chalcone and flavone moieties in the biflavonoid structure through the correlation of H-2″ (δ_H_ 4.57) to the carbons C-3 (δ_C_ 119.4) and C-4 (δ_C_ 181.1); the hydrogen H-3″ (δ_H_ 3.44 and 2.97) to the carbon C-3 (δ_C_ 119.4) confirmed the structure of a biflavonoid (Figure 9). The NMR data were analogous to those of caesalpinioflavone [24], except for the presence of resonances associated with the methoxyl substituent (δ_H_ 3.66/δ_C_ 55.4). Thus, compound **2** was identified as an novel *O*-methoxylated derivative of the biflavonoid caesalpinioflavone, named 4‴-methoxycaesalpinioflavone. The assignments of all carbons and their respective hydrogens are presented in Table 2.

The HRESIMS spectrum (Supplementary Materials) obtained in negative mode for compound **3** exhibited [M − H]^−^ at *m*/*z* 539.1356 (calcd. for C_31_H_23_O_9_, 539.1342). All spectra (Supplementary Materials) obtained in DMSO-*d6* were similar to compound **2**, and it was found that this is another new *O*-methoxylated derivative of biflavonoid caesalpinioflavone. The only difference is the position of the methoxyl group (δ_H_ 3.80) which is bound to the C-7 (δ_C_ 165.9). Thus, such a substance may be referred to as 7-methoxycaesalpinioflavone (**3**). The assignments of all carbons and their respective hydrogens are presented in Table 3.

Compounds **1** and **4** was identified as caesalpinioflavone and rhuschalcone VI, respectively, through the comparison of NMR data with those reported in the literature [24,34].

## 3. Materials and Methods

### 3.1. Equipments, Reagents and Solvents

NMR spectra were obtained at 25 °C, with a 300 MHz (^1^H) and 75 MHz (^13^C) Bruker spectrometer (300 MHz, Billerica, MA, USA). Chemical shifts were referenced to the residual DMSO-*d_6_* signals (δ_H_2.54 and δ_C_ 39.5). HRMS analyses were performed with an ESI-TOF on a Waters-XEVO-G2XSQTOF–LC system mass spectrometer (Milford, MA, USA). HPLC analyses were performed using a Shimadzu (Quioto, Japan) Prominence modulated equipment with two mobile phase pumps LC-20AD and DGU-20A 3R degasser, auto-injector SIL-20A HT, oven for column CTO-20A, UV-Vis detector with diode arrangement (SPD-M20A, DAD), and CBM-20a communicator. The samples were also registered at 210 and 254 nm. All solvents used were HPLC grade and Milli-Q water.

### 3.2. Plant Material and Preparation of Extracts

The plant material (stem bark, flowers, and leaves of *P. pluviosa*) was collected in March 2016 on the campus of Federal University of Alfenas, Unifal-MG in the city of Alfenas-MG. The geographical coordinates: latitude: 21°25′45″ south and longitude: 45°56′50″ west. The botanical identification was carried out at the Federal University of Alfenas by Professor Dr. Marcelo Polo (Institute of Natural Sciences), and the species was catalogued in a voucher (UALF-1634), which was deposited in the herbarium of the UNIFAL-MG.

The plant material was submitted to oven drying process with air circulation for 72 h at standard temperature (45 °C), powdered in TE-625 knife mill. The dried and ground plant material was submitted to exhaustive maceration for four days at room temperature in EtOH. The crude extract was concentrated under reduced pressure at 40 °C, resuspended in EtOH/H_2_O (3:1), and successively partitioned using hexane and EtOAc. These procedures resulted in hexane, EtOAc and hydroethanolic fractions of flowers (FlHe, FlAc, and FlE, respectively), leaves (FoHe, FoAc, and FoE, respectively) and stems bark (CaHe, CaAc, and CaE, respectively) of *P. pluviosa*.

### 3.3. Isolation of Compounds ***1**–**4***

The dried and ground stem bark (3 kg) of *P. pluviosa* was submitted to exhaustive maceration for four days using a mixture of EtOAc and hexane in the ratio of 1:1 (5 L). This material was then concentrated under reduced pressure and at 40 °C, yielding in 110 g of the ethyl acetate/hexane extract from *P. pluviosa* stem bark (CaAcHe). An aliquot of the CaAcHe extract (10.0 g) was subjected to separation by chromatography on silica gel (0.063–0.200 mm, 271.5 g), and the gradient 100% hexane, 1:1 hexane/EtOAc, 100% EtOAc, 1:1 EtOAc/EtOH, and 100% EtOH as eluent, affording four fractions. Fraction 2 (3.0 g), eluted with 1:1 hexane/EtOAc, was subjected to Sephadex LH-20 (Sigma-Aldrich, San Luis, MI, USA) eluted with MeOH to yield compound **4** (28.0 mg). Fraction 3 (2.5 g), eluted with 100% EtOAc, was subjected to silica gel (Merck, Darmstadt, Germany) column chromatography eluted with 1:1 hexane/EtOAc, 4:6 hexane/EtOAc, 100% EtOAc, and 100% EtOH to yield fours groups (A–D). Group C (100 mg), eluted with 100% EtOAc, was purified by reversed-phase HPLC using a column Inertsil ODS-4 (5 μm, 7.6 mm × 250 mm, GL Science, Japan) eluted with 50% ACN:H_2_O with 0.1% HOAc for 30 min, with a flow rate of 1.5 mL/min to yield compounds **2** (13.0 mg) and **3** (5.0 mg). Compound **1** was obtained according to the method previously described [24].

### 3.4. Anti-Inflammatory Bioassay

Animals were supplied by the facilities of UNIFAL-MG and kept under standard laboratory conditions (21–24 °C on a 12 h light-dark cycle), with water and food ad libitum. Male Swiss mice (25–35 g) were used in groups of eight animals. Experiments were approved by the Ethics Committee on Animal Use (CEUA) of UNIFAL-MG (approval protocol number 02/2017), which followed rules of the National Council for Control of Animal Experimentation (CONCEA). Cutaneous inflammation was induced in the left ear of the mice (*n* = 8) by topical application of 20 μL of the irritant solution of croton oil (5% *v*/*v*) dissolved in acetone. Only acetone was applied in the right ear. Thirty minutes after the application of croton oil, topical treatment with hexane, EtOAc, and hydroethanolic fractions of flowers, leaves, and bark of *P. pluviosa* were applied at a dose of 0.5 mg/ear. Samples were dissolved in 20% acetone and glycerol. The isolated compounds **1**, **2**, and **4** were similarly evaluated. Animals from the negative control group received only the irritant solution, while those in the positive control group were topically treated with reference drugs indomethacin and dexamethasone, also at doses of 0.5 mg/ear, to control the inhibition of edema and neutrophil recruitment, respectively. Animals were euthanized by inhalation of isoflurane six hours after the induction of inflammation for excision of ear fragment (6 mm diameter) from both ears using a punch. The quantification of edema was determined by means of the weighting difference between the fragments of left and respective right ears. Data were analyzed by one-way ANOVA followed by Dunnett’s multiple comparison test expressed as mean ± SEM (standard error). The percentage of edema inhibition was calculated as (vehicle-treatment/vehicle) × 100. Statistical analysis of the data was performed in GraphPad Prism Version 6.0 (Microsoft, Redmond, WA, USA). The obtained ear fragments were maintained in 200 μL of NaEDTA/NaCl pH 4.7 buffer at −20 °C until analysis, with handling was always performed under ice.

### 3.5. Procedure for Neutrophil Recruitment

Ear fragments, in buffer solution, were transferred to a test tube, to which an additional 400 μL NaEDTA/NaCl pH 4.7 buffer was added. These were homogenized using the Turrax tissue macerator (13,000 rpm, three times) and then centrifuged at 3000× *g* for 15 min at 4 °C. The supernatant was removed and the precipitate was resuspended in 600 μL of pH 5.4 buffer containing 0.5% hexadecyltrimethyl ammonium bromide, and homogenized again using the Turrax tissue macerator 13,000 rpm (three times). Samples were centrifuged at 10,000× *g* for 15 min at 4 °C and the supernatants were used to evaluate inhibition of the MPO enzyme. For the quantification of MPO, 50 μL of supernatant from each sample was added 96-well plates in triplicates. On each plate there was a replicate of each control (positive and negative). All samples were handled at low temperatures and under minimum light. After plating samples, plates were kept capped inside the refrigerator. A solution was prepared with reagents A and B (1.6 mM tetramethylbenzidine and 0.5 mM H_2_O_2_) from the BD OptEIATM kit in the ratio of 1:1, a total of 6 mL solution per plate was used, 15 min prior analysis. Then, 50 μL of the prepared solution was added to each well, and after 10 min, 50 μL of the aqueous solution of sulfuric acid was added to stop the enzymatic reaction. After further 10 min, the absorbance was read at 450 nm. The results were analyzed by one-way ANOVA followed by Dunett’s multiple comparison test expressed as mean ± standard error (SEM). The program used for statistical analysis of the data was GraphPad Prism Version 6.0.

### 3.6. Chemical and Metabolomics Analysis

All fractions were evaluated in UHPLC-UV-HRFTMS (Thermo Scientific Exactive™ equipped with Orbitrap™ technology, Thermo Scientific^®^, Waltham, MA, USA). The following chromatography method was used: ACN/H_2_O gradient; 0.1% formic acid in water, 5% ACN 5 min, 5–100% ACN 50 min; 3 μm particle diameter chromatography column (C_18_- 150 × 3 mm; ACE^®^, Aberdeen, Scotland), and flow of 0.3 mL/min. The column temperature was controlled at 30 °C. The spectrometer was operated with the following parameters: concomitant positive and negative mode; scanning range of 75–1200 *m*/*z*; high resolution: 50,000; microbeam: one; locking mass (known *m*/*z* ion in the samples, allowing real-time correction to be made of *m*/*z* reading deviations; 83.0604 *m*/*z* of the ACN + H dimer) in the positive mode; maximum injection time: 250 ms. The parameters of the ESI ionization source were: gas flow rate: 50; auxiliary gas flow rate: 17; Spray tension: + and −4.5 kV; spray stream: 1.4 MA; capillary temperature: 320 °C; capillary voltage: + and −30 V; lens voltage: + and −90 V; cone tension (skimmer): + and −20 V. Prior to the analysis sequence, a calibration of the apparatus was performed for each polarity using low molecular weight contaminants and Thermo calmix solution (Sigma-Aldrich^®^). Data were recorded using the Xcalibur 2.1.0 software (Thermo Fisher Scientific©). Samples were randomly analyzed, with one sample and one blank each being analyzed at the beginning, in the middle, and at the end of the chromatographic analysis sequence. Using the software MZmine 3.0 (MZmine Development Team, Free Software Foundation Inc., Boston, MA, USA) the chromatograms of the different fractions were deconvoluted, their isotopes eliminated, identical peaks in the different fractions aligned, and gaps filled. From the results of the evaluation of the anti-inflammatory activity and the chromatographic data of each fraction (previously treated in MZmine and exported from this software in .csv format), metabolomics analyses were performed. These analyses were performed using supervised multivariate statistical analyses in the SIMCA-P 13.0.2.0 © (Umetrics, Umeå, Sweden) software to determine biomarkers of the evaluated activity. PCA and PLS-DA were performed in SIMCA-P, and the parameters used were set by default. The data were evaluated with and without normalization; the ParN normalization was selected, which generated the best statistical model. Since the PCA (Supplementary Materials) model was well fitted (R2X = 0.53) and did not indicate bias, we performed PLS-DA, based on a discriminant analysis due to the presence of a class variable (Y variable). Afterwards, the important variable for the projection (VIP) and the general view of the coefficients were used for the identification of biomarkers for the anti-inflammatory properties of the fractions of *P. pluviosa*. Validation values R2X = 0.617, R2Y = 0.993, and Q2 = 0.752. The VIPs obtained from the PLS-DA model were then dereplicated using the comprehensive high-resolution mass dictionary, the Dictionary of Natural Products© (DNP). A comparison with literature data was performed by the construction of an in-house database from the chemical constituents already described in the literature for the genus *Poincianella*. This database was built by downloading the monoisotopic mass information from SciFinder Scholar^®^.

## 4. Conclusions

The present work intended to use metabolomics strategies along with multivariate statistical analysis to rationally isolate compounds of interest for their anti-inflammatory activities. Compounds isolated through these approaches were elucidated and the values of their biological assays showed that they are indeed effective against inflammatory processes. This work also relates, for the first time, the effect of novel compounds 4‴-methoxycaesalpinioflavone, 7-methoxycaesalpinioflavone, as well as known derivatives rhuschalcone VI and caesalpinioflavone, on the inhibition of edema and neutrophil recruitment. Results showed that two of them presented better efficacies than the reference drugs (indomethacin and dexamethasone). In this sense, the experimental results corroborated the use of metabolomics and statistical analyses guiding the isolation of promising natural products.

## Figures and Tables

**Table 1:** Variables important for the projection (VIPs) of the PLS-DA model built for the anti-inflammatory activity of fractions of *P. pluviosa*.

ID	VIP	*m*/*z*	Rt	Error (ppm)	Molecular Formula [M − H]^−^	Hits
43 *	1.8	329.030087	10.6	−0.62	C_16_H_9_O_8_	11 (phenolic compounds)
31 *	1.4	525.118560	16.8	−2.71	C_30_H_21_O_9_	22 (caesalpinioflavone, **1**)
28 *	0.9	509.123606	18.5	−2.24	C_30_H_21_O_8_	22 (rhuschalcone VI, **4**)
100 *	1.5	539.134155	19.1	−2.26	C_31_H_23_O_9_	1 (7-methoxycaesalpinioflavone, **3**)
6 *	1.3	539.134196	19.9	−2.26	C_31_H_23_O_9_	1 (4‴-methoxycaesalpinioflavone,**2**)
231 *	1.1	271.227588	24.1	−1.43	C_16_H_31_O_3_	17 (fatty acids)
457	2.2	433.113306	10.0	0.40	C_21_H_26_O_3_N_2_Br	0
188	1.2	319.045785	10.6	−0.50	C_15_H_11_O_8_	13 (ethyl brevifolincarboxylate)
491	2.6	395.161091	11.1	−0.37	C_22_H_23_O_5_N_2_	0
352	1.8	237.040059	11.1	−1.31	C_11_H_9_O_6_	18 (phenolic compounds)
402	1.2	262.071942	11.3	−0.59	C_13_H_12_O_5_N	0
502	2.1	425.171936	11.6	0.17	C_23_H_25_O_6_N_2_	0
489	2.9	379.165825	12.0		C_22_H_23_O_4_N_2_	0
488	3.0	409.176757	12.4	−0.26	C_23_H_25_O_5_N_2_	0
508	1.7	354.098236	12.7	−0.21	C_19_H_16_O_6_N	0
509	1.9	439.187347	12.8	−0.26	C_24_H_27_O_6_N_2_	0
309	1.2	571.088124	13.1	−0.13	C_30_H_19_O_12_	14 (phenolic compounds)
190	3.8	285.040309	13.2	−1.16	C_15_H_9_O_6_	10 (phenolic compounds)
403	1.2	391.066808	13.2	−0.67	C_18_H_15_O_10_	3 (phenolic compounds)
512	1.2	402.249603	13.9	−0.30	C_20_H_36_O_7_N	0
520	1.3	437.231079	14.4	−0.17	C_21_H_38_O_7_Cl	0
486	2.1	543.129247	14.7	−0.78	C_30_H_23_O_10_	19 (phenolic compounds)
301	1.8	269.045442	14.7	0.27	C_15_H_9_O_5_	103 (phenolic compounds)
504	1.2	655.442627	15.3	−0.07	C_36_H_63_O_10_	0
181	3.6	327.217286	15.4	−1.37	C_18_H_31_O_5_	37 (phenolic compounds)
475	2.5	363.193962	15.4	−1.20	C_18_H_32_O_5_Cl	0
494	1.7	329.066286	15.9	−1.18	C_17_H_13_O_7_	153 (phenolic compounds)
392	6.9	271.061001	16.1	−1.39	C_15_H_11_O_5_	115 (phenolic compounds)
76	1.8	329.233110	16.3	−0.69	C_18_H_33_O_5_	22 (fatty acids)
528	1.2	323.199127	16.7	−1.03	C_16_H_32_O_4_Cl	0
506	1.9	365.209715	17.2	−0.85	C_18_H_34_O_5_Cl	0
505	2.0	403.233625	17.5	−0.25	C_20_H_35_O_8_	0
487	2.1	387.238416	18.7	−1.02	C_20_H_35_O_7_	1 (norcaperatic acid)
490	1.8	309.082260	2.1	−1.49	C_11_H_17_O_10_	1 (glucopyranosyl)
451	1.3	331.066886	2.3	−0.55	C_13_H_15_O_10_	12 (phenolic compounds)
419	1.2	433.235646	21.2	2.85	C_23_H_33_O_6_N_2_	0
205	6.6	611.408728	22.4	−0.41	C_35_H_60_O_6_Cl	0
496	2.3	621.437117	22.4	−0.29	C_36_H_61_O_8_	11 (phenolic compounds)
485	4.1	613.406587	22.6	−0.58	C_32_H_57_O_9_N_2_	0
534	1.4	734.517439	22.6	0.28	C_37_H_72_O_11_N_3_	0
495	2.6	750.529089	24.0	1.30	C_40_H_82_O_2_N_3_BrCl	0
466	1.2	561.429542	25.8	0.74	C_32_H_62_O_5_Cl	0
282	1.4	748.513773	26.2		C_41_H_70_O_9_N_3_	0
517	1.8	830.114318	8.3	0.01	C_30_H_24_O_20_N_9_	0
206	2.3	951.074661	8.4	−1.21	C_42_H_23_O_23_N_4_	0
359	1.2	275.019645	8.5	−0.39	C_13_H_7_O_7_	3 (xanthones)
526	1.7	476.040832	8.6	0.62	C_19_H_14_O_10_N_3_S	0
492	2.6	337.092738	9.2	−0.27	C_16_H_17_O_8_	18 (phenolic compounds)
185	2.6	300.998685	9.3	−1.10	C_14_H_5_O_8_	1 (ellagic acid)
493	2.3	252.050914	9.6	−1.96	C_11_H_10_O_6_N	0
180	2.8	463.087903	9.9	−1.60	C_21_H_19_O_12_	71 (phenolic compounds)

* Positive coefficient correlation value to both inhibition group (Figure 3).

**Table 2:** NMR data for 4‴-methoxycaesalpinioflavone, **2** (in DMSO-*d_6_*).

Position	δ_C_, Type	δ_H_, mult. (*J* in Hz)
2	164.4, C	-
3	119.4, C	-
4	181.1, C	-
5	161.9, C	-
6	103.5, CH	6.17 d (2.0)
7	165.2, C	-
8	94.1, CH	6.26 d (2.0)
9	157.7, C	-
10	99.4, C	-
1′	122.8, C	-
2′/6′	130.7, CH	7.08 d (8.7)
3′/5′	115.7, CH	6.81 d (8.7)
4′	160.0, C	-
2″	48.4, CH	4.57 t (6.7)
3a″	34.5, CH	2.97 dd (13.9, 6.7)
3b″	34.5, CH	3.44 (m)
4″	201.3, C	-
5″	131.7, CH	7.18 d (8.6)
6″	108.2, CH	6.15 dd (8.6, 2.2)
7″	163.5, C	-
8″	103.0, CH	6.17 d (2.2)
9″	164.4, C	-
10″	113.1, C	-
1‴	132.2, C	-
2‴/6‴	114.0, CH	6.73 d (8.6)
3‴/5‴	130.4, CH	6.98 d (8.6)
4‴	158.1, C	-
4‴-OCH_3_	55.4	3.66 s
OH	-	11.87 s
OH	-	12.66 s

**Table 3:** NMR data for compound 7-methoxycaesalpinioflavone, **3** (in DMSO-*d_6_*).

Position	δ_C_, Type	δ_H_, mult. (*J* in Hz)
2	164.6, C	-
3	119.7, C	-
4	181.4, C	-
5	157.6, C	-
6	92.7, CH	6.57 d (2.0)
7	165.9, C	-
8	98.6, CH	6.36 d (2.0)
9	161.6, C	-
10	104.6, C	-
1′	122.7, C	-
2′/6′	130.7, CH	7.07 d (8.7)
3′/5′	115.7, CH	6.81 d (8.7)
4′	160.1, C	-
2″	48.6, CH	4.55 t (6.9)
3a″	34.5, CH	2.98 dd (13.9, 7.14)
3b″	34.5, CH	3.35 (m)
4″	201.2, C	-
5″	131.7, CH	7.17 d (8.6)
6″	108.3, CH	6.15 dd (9.4, 2.2)
7″	163.5, C	-
8″	103.1, CH	6.16 d (2.2)
9″	164.8, C	-
10″	113.1, C	-
1‴	130.3, C	-
2‴/6‴	130.4, CH	6.85 d (8.6)
3‴/5‴	115.4, CH	6.56 d (8.6)
4‴	156.0, C	-
7-OCH_3_	56.6	3.80 s
OH	-	11.85 s
OH	-	12.69 s

## Supplementary Materials

The following are available online. Figures S1–S14: NMR and MS spectra of compounds **1–4**, Figure S15: PCA of *P. pluviosa* fractions. Tables S1–S4: Anti-inflammatory data.
