# Supplementary material for: Anti-Inflammatory Derivatives with Dual Mechanism of Action from the Metabolomic Screening of Poincianella pluviosa
Source: Molecules. 2019 Nov 29;24(23):4375. doi: 10.3390/molecules24234375 (PMC6930619; doi:10.3390/molecules24234375)
Supplement: Supplementary file 1 [file molecules-24-04375-s001.pdf]

Supporting Information

For

Anti-Inflammatory Derivatives with Dual Mechanism of Action from the Metabolomic Screening of *Poincianella pluviosa*

Olívia da S. Domingos [1], Bianca G. V. Alcântara [1], Mário F. C. Santos [1], Tatiane C. S. Maiolini [1], Danielle F. Dias [1], João L. Baldim [1,2], João Henrique G. Lago [3], Marisi G. Soares [1,*] and Daniela A. Chagas-Paula,[1,*]

1 Instituto de Química–Universidade Federal de Alfenas, MG, Brazil; oliviadomingos@hotmail.com (O.d.S.D.); bianca.vasconcelos.alcantara@gmail.com (B.G.V.A.); mariosantos408@gmail.com or marisigs@gmail.com (M.F.C.S.); tatychryss@gmail.com (T.C.S.M.); danielle.dias@unifal-mg.edu.br (D.F.D.)

2 Instituto Federal de de Educação, Ciência e Tecnologia do Sul de Minas Gerais - IFSULDEMINAS, MG, Brazil jotaelebaldim@gmail.com (J.L.B.)

3 Centro de Ciências Naturais e Humanas, Universidade Federal do ABC, SP, Brazil; joao.lago@ufabc.edu.br or joaohglago@gmail.com

* Correspondence: marisigs@gmail.com (M.G.S.); da.chagaspaula@gmail.com (D.A.C.-P.); Tel.: +55-11-37019713 (M.G.S. &D.A.C.-P.)

Contents:

Page S2. Table S1. Effect of fractions of *C. pluviosa* on ear edema exhibited in percentage of inhibition.
Page S3. Table S2. Effect of fractions of *C. pluviosa* on neutrophil recruitment via myeloperoxidase dosing (MPO).
Page S4. Table S3. Percentage of inhibition of ear edema by isolated compounds.
Page S5. Table S4. Effect of compounds on neutrophil recruitment measured via myeloperoxidase quantification (MPO).
Page S6. Figure S1. ^{1}H NMR (300 MHz, DMSO-d_6) spectrum of the compound (**2**).
Page S7. Figure S2. ^{13}C NMR (75 MHz, DMSO-d_6) spectrum of the compound (**2**).
Page S8. Figure S3. COSY (DMSO-d_6) spectrum of the compound (**2**).
Page S9. Figure S4. HSQC (DMSO-d_6) spectrum of the compound (**2**).
Page S10. Figure S5. HMBC (DMSO-d_6) spectrum of the compound (**2**).
Page S11. Figure S6. ^{1}H NMR (300 MHz, DMSO-d_6) spectrum of the compound (**3**).
Page S12. Figure S7. ^{13}C NMR (75 MHz, DMSO-d_6) spectrum of the compound (**3**).
Page S13. Figure S8. COSY (DMSO-d_6) spectrum of the compound (**3**).
Page S14. Figure S9. HSQC (DMSO-d_6) spectrum of the compound (**3**).
Page S15. Figure S10. HMBC (DMSO-d_6) spectrum of the compound (**3**).
Page S16. Figure S11. ^{1}H NMR (300 MHz, CD_3OD) spectrum of the compound (**4**).
Page S17. Figure S12. Mass spectrum of the compound (**2**).
Page S18. Figure S13. Mass spectrum of the compound (**3**).
Page S19. Figure S14. Mass spectrum of the compound (**4**).
Page S20. Figure S15. Principal component analysis (PCA) of samples from *P. pluviosa*.

Table S1. Effect of fractions of *C. pluviosa* on ear edema exhibited in percentage of inhibition. The results were analyzed by one-way ANOVA followed by Dunett's multiple comparison test and expressed as mean ± SEM. * $p \leq 0.05$ compared to vehicle , ^{ns} no significant difference compared with the vehicle ($P > 0.05$) and # $p \leq 0.05$ compared to the indomethacin.

Sample	Edema (mg) Mean ± SD	% inhibition
CaHe	$0.66 \pm 0.6^{*}$	82.53
CaAc	$1.30 \pm 0.7^{*}$	65.61
CaE	$2.09 \pm 1.1^{*}$	44.71
FoHe	$1.46 \pm 0.5^{*}$	61.38
FoAc	$1.11 \pm 0.4^{*}$	70.64
FoE	$1.10 \pm 0.4^{*}$	70.90
FlHe	$1.99 \pm 1.0^{*}$	47.35
FlAc	$3.44 \pm 1.2^{ns,\#}$	8.99
FlE	$2.05 \pm 1.0^{*}$	45.77
Vehicle	3.78 ± 0.5	-
Indomethacin	$1.64 \pm 0.4^{*}$	56.61
Dexamethasone	$0.51 \pm 0.3^{*,\#}$	86.51

Table S2. Effect of fractions of *C. pluviosa* on neutrophil recruitment via myeloperoxidase dosing (MPO). The results were analyzed by one-way ANOVA followed by Dunett's multiple comparison test and expressed as mean ± SEM. * p≤ 0.05 compared to the vehicle and # p≤ 0.05 compared to the dexamethasone.

Sample	Absorbance<br>Mean ± SD
CaHe	$0.52 \pm 0.18^{*}$
CaAc	$0.59 \pm 0.20^{*}$
CaE	$0.52 \pm 0.13^{*}$
FoHe	$0.47 \pm 0.18^{*}$
FoAc	$0.54 \pm 0.16^{*}$
FoE	$0.48 \pm 0.15^{*}$
FlHe	$0.59 \pm 0.16^{*}$
FlAc	$0.54 \pm 0.13^{*}$
FlE	$0.60 \pm 0.16^{*}$
Vehicle	$0.86 \pm 0.05^{\#}$
Dexamethasone	$0.53 \pm 0.11^{*}$

Table S3. Percentage of inhibition of ear edema by isolated compounds. The results were analyzed by one-way ANOVA followed by Dunett's multiple comparison test and expressed as mean ± SEM. * p≤ 0.05 compared to the vehicle and # p≤ 0.05 compared to the dexamethasone.

Sample	Edema (mg) Mean ± SD	% inhibition
Caesalpinioflavone (**1**)	$1.0 \pm 0.7^{*,\#}$	69.23
4’”-metoxi-caesalpinioflavone (**2**)	$1.71 \pm 0.9^{*}$	47.38
Rhuschalcone VI (**4**)	$1.85 \pm 0.4^{*}$	43.07
Vehicle	$3.25 \pm 0.8^{\#}$	-
Indomethacin	$1.84 \pm 0.8^{*}$	43.38
Dexamethasone	$1.43 \pm 0.8^{*}$	56.00

Table S4. Effect of compounds on neutrophil recruitment measured via myeloperoxidase quantification (MPO). The results were analyzed by one-way ANOVA followed by Dunett's multiple comparison test and expressed as mean ± SEM. * $p \leq 0.05$ compared to vehicle and # $p \leq 0.05$ compared to the dexamethasone.

Sample	Absorbance Mean ± SD
Caesalpinioflavone (**1**)	$0.48 \pm 0.09^{*}$
4’”-metoxi-caesalpinioflavone (**2**)	$0.60 \pm 0.15^{*,\#}$
Rhuschalcone VI (**4**)	$0.16 \pm 0.05^{*,\#}$
Vehicle	$0.95 \pm 0.07^{\#}$
Dexamethason	$0.54 \pm 0.03^{*}$

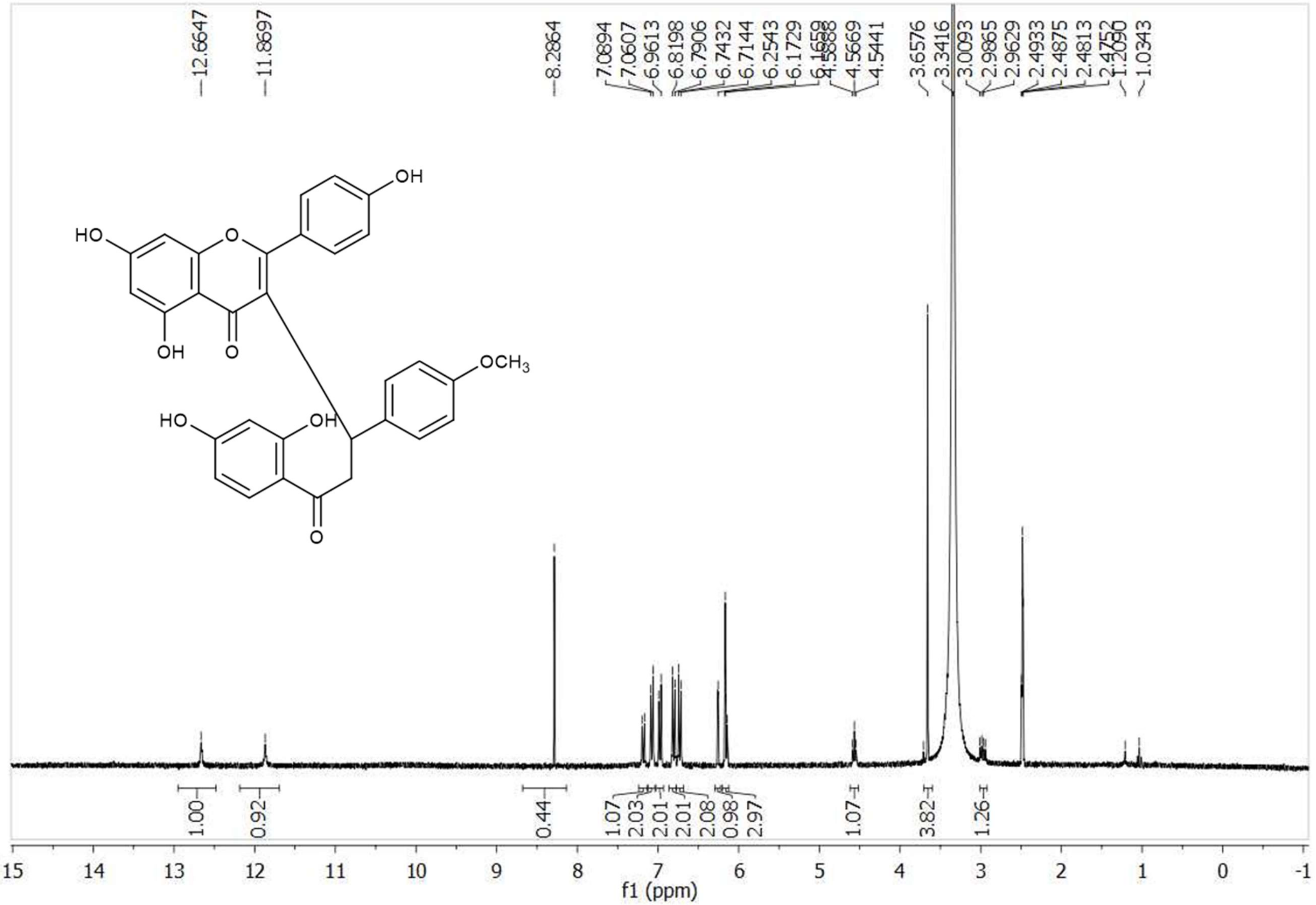

Figure S1. ^{1}H NMR (300 MHz, DMSO-d_6) spectrum of the compound (**2**).

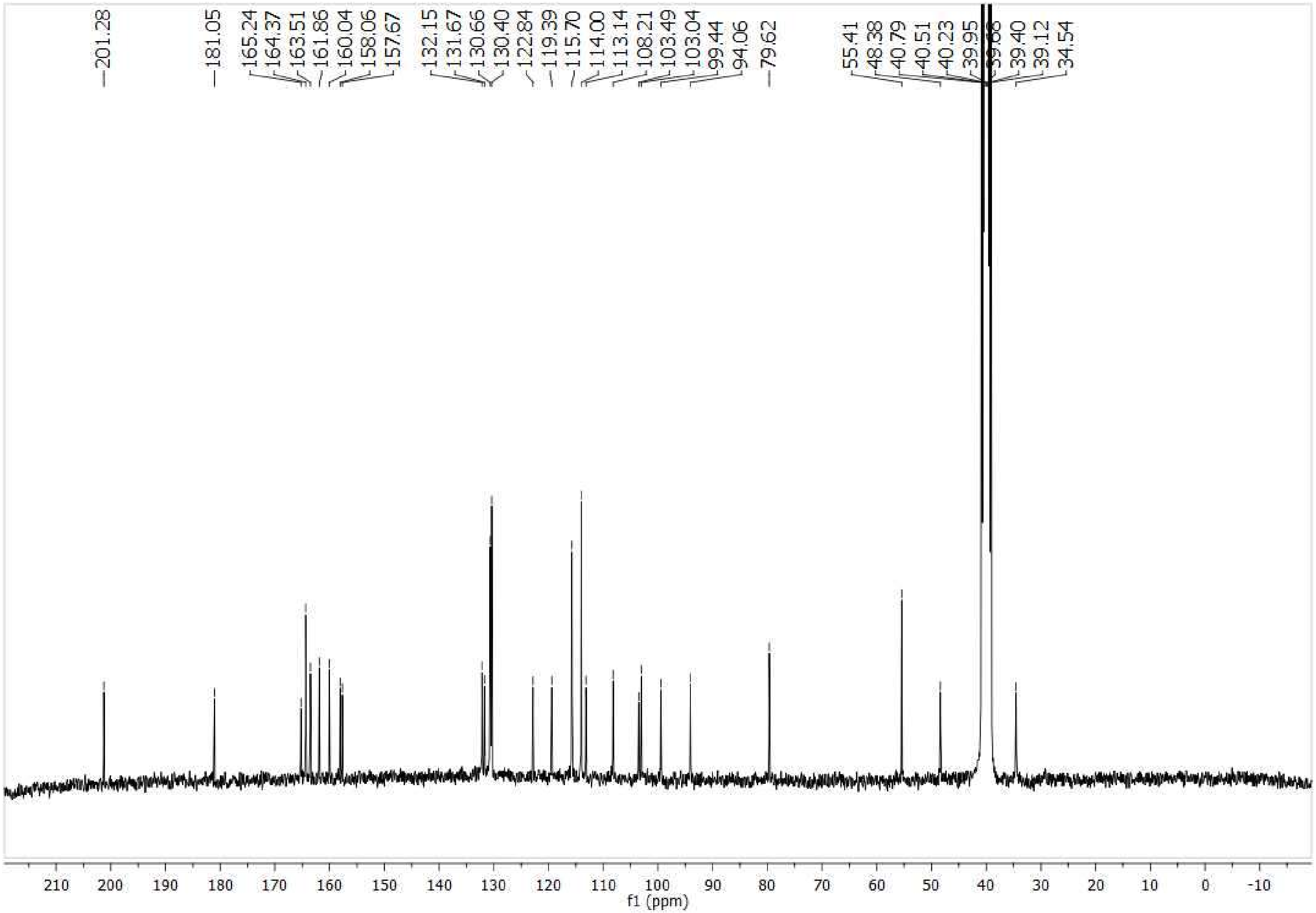

Figure S2. ^{13}C NMR (75 MHz, DMSO-d_6) spectrum of the compound (**2**).

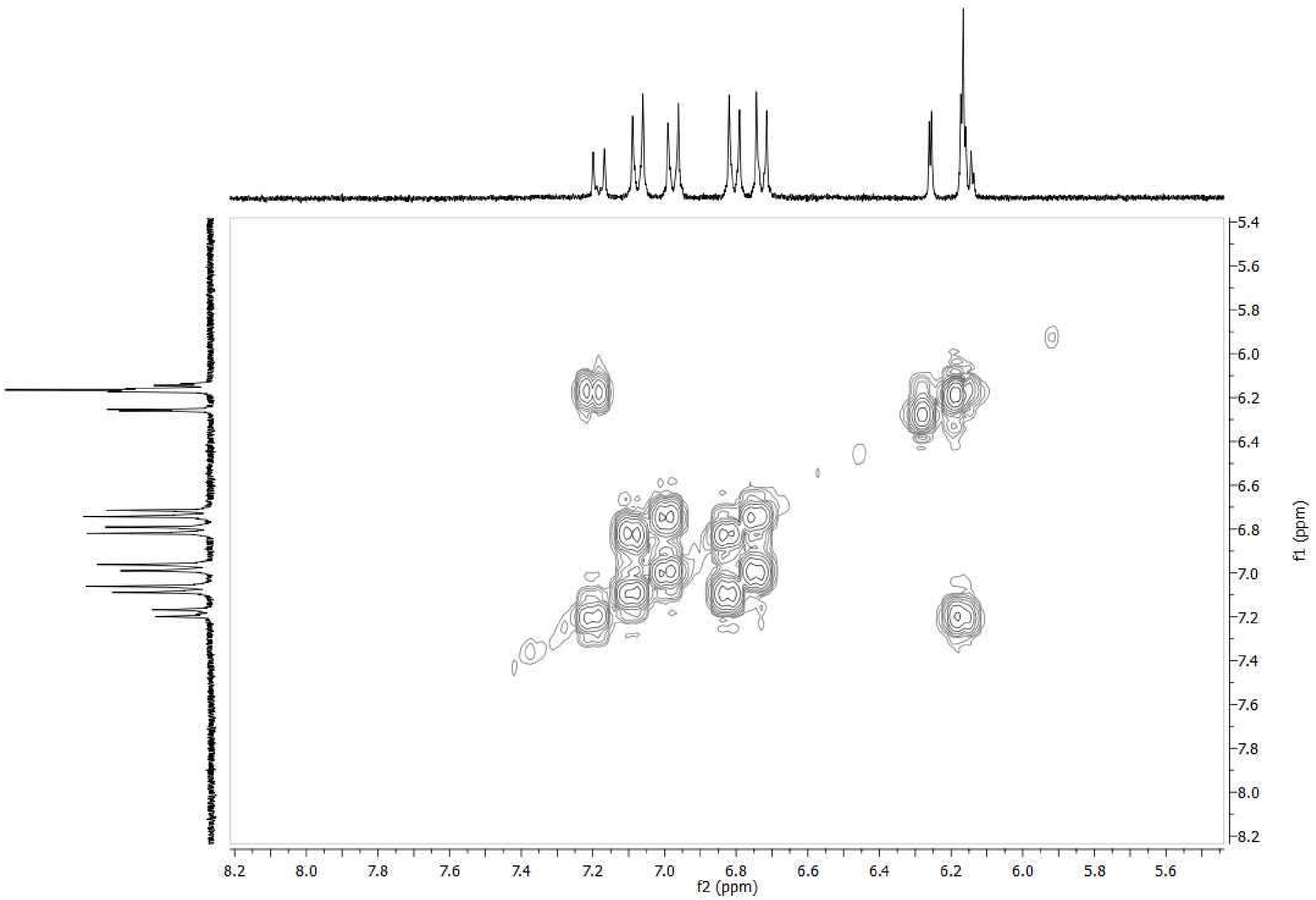

Figure S3. COSY (DMSO-d_6) spectrum of the compound (**2**).

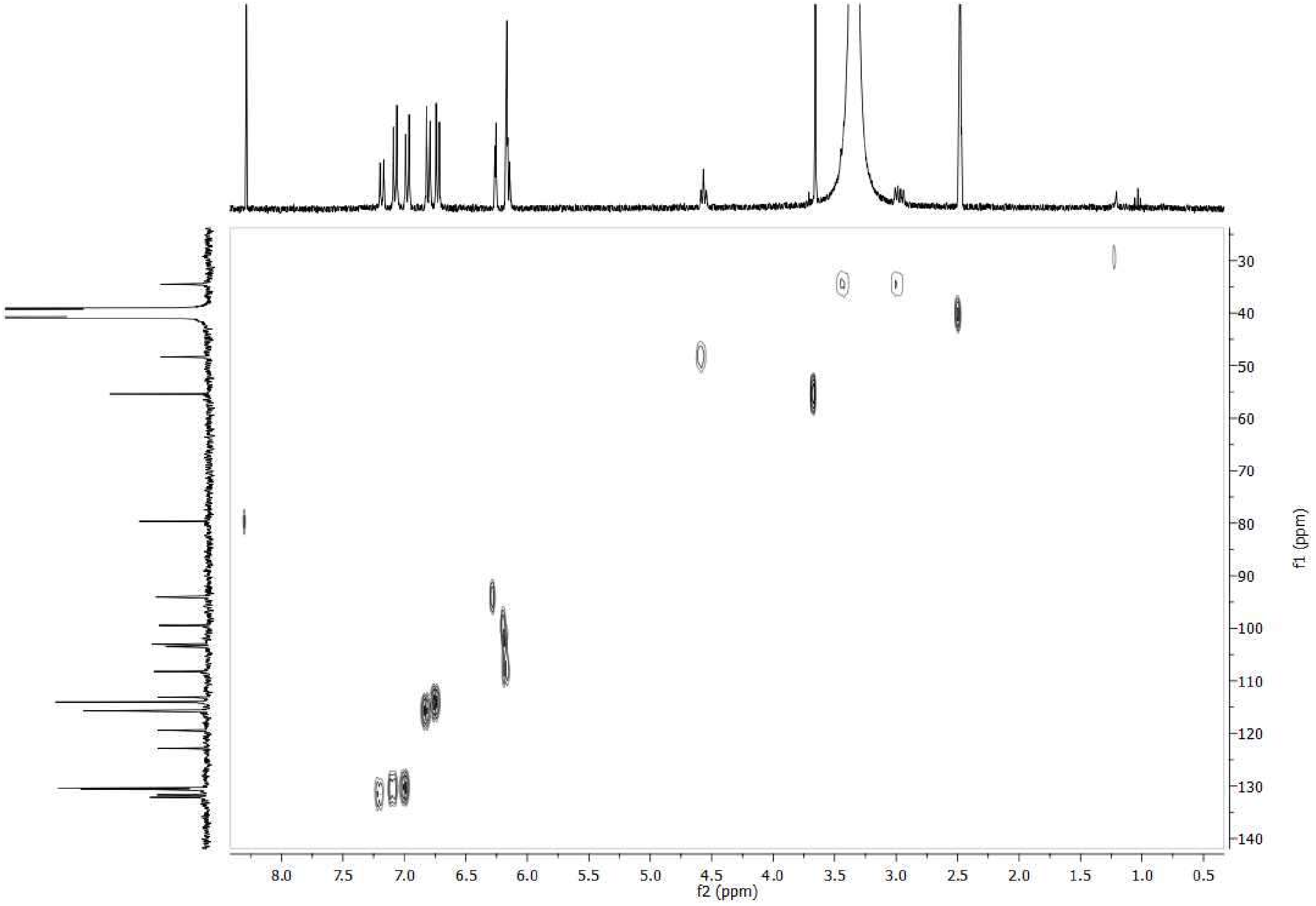

Figure S4. HSQC (DMSO-d_6) spectrum of the compound (**2**).

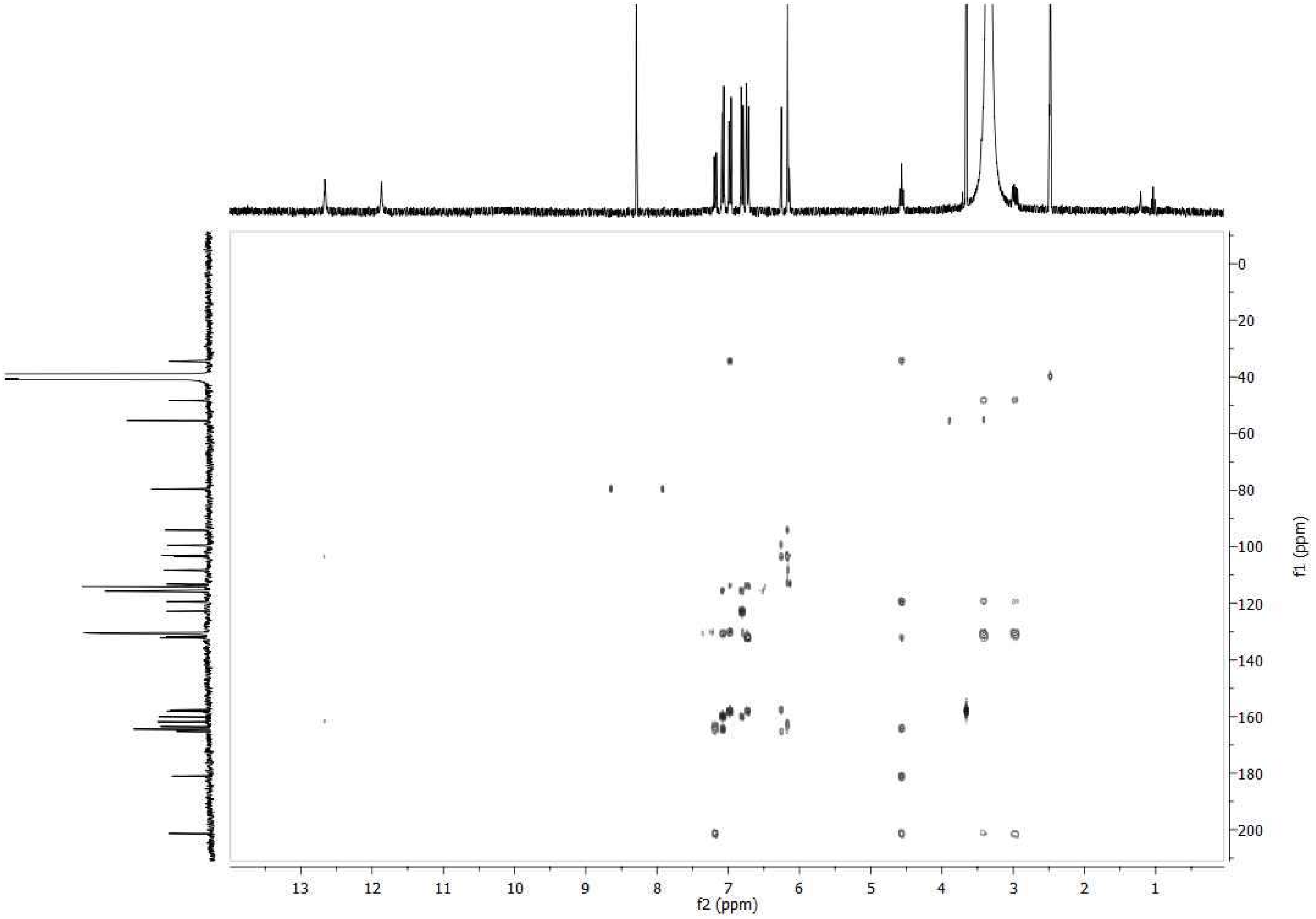

Figure S5. HMBC (DMSO-d_6) spectrum of the compound (**2**).

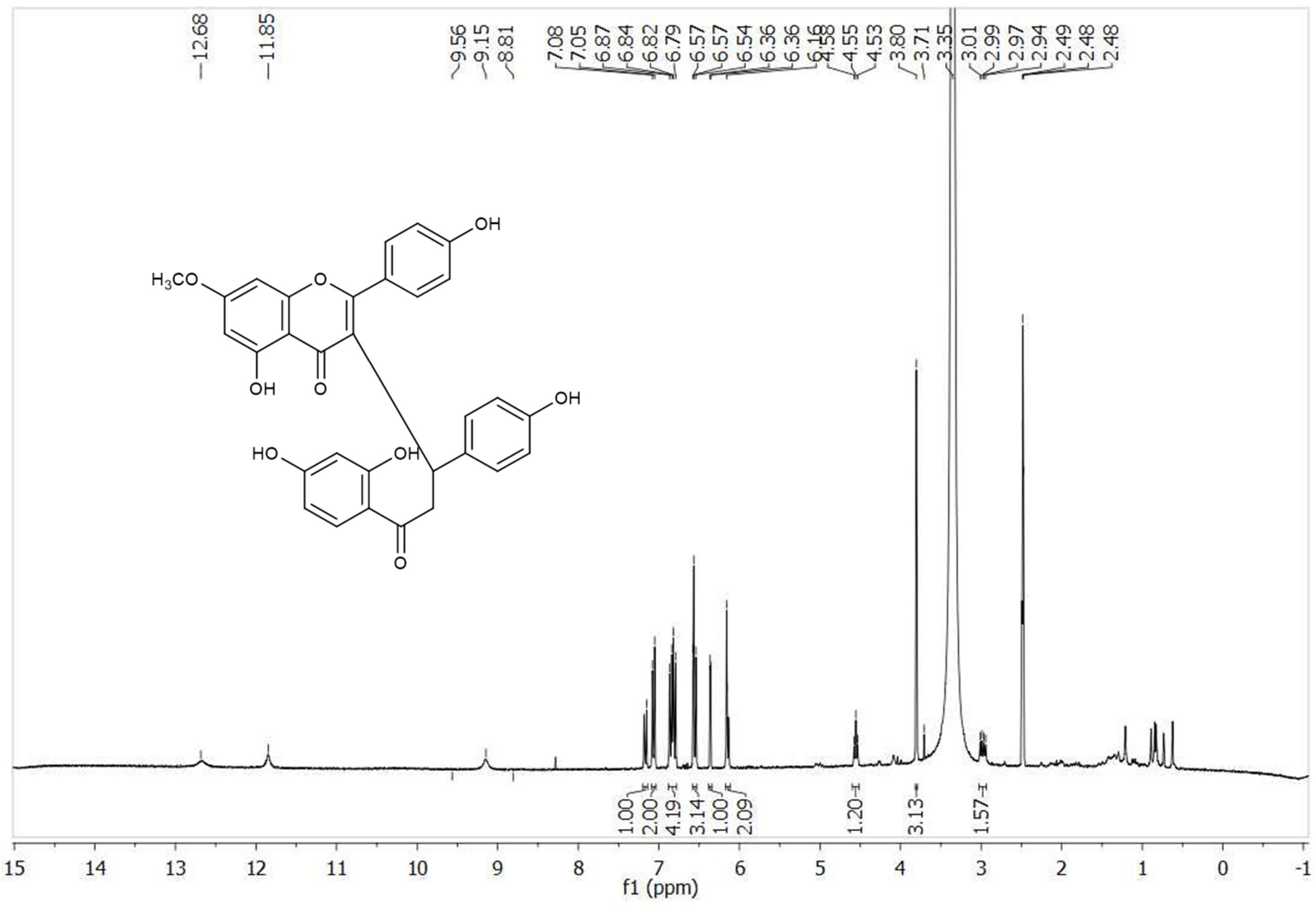

Figure S6. ^{1}H NMR (300 MHz, DMSO-d_6) spectrum of the compound (**3**).

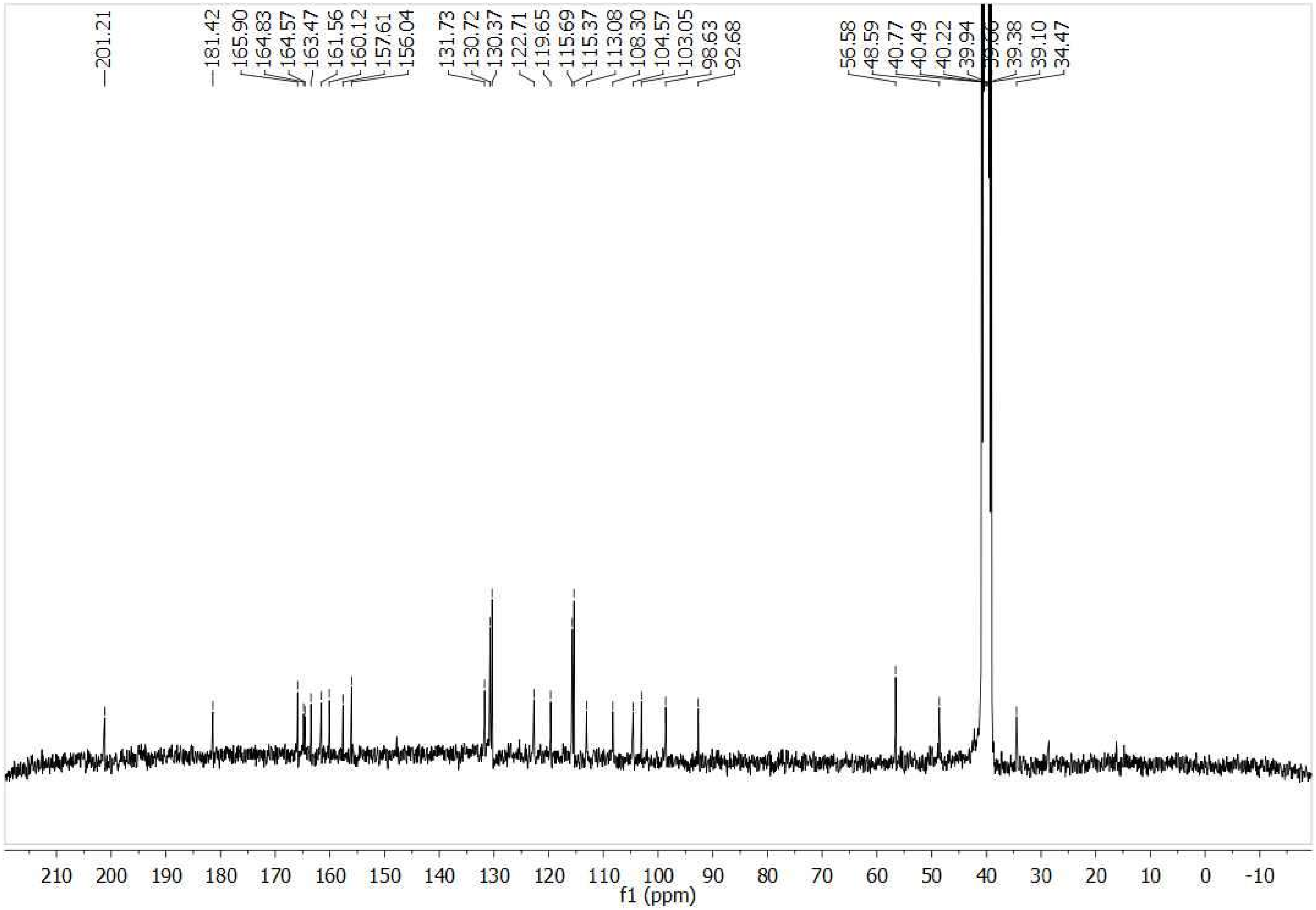

Figure S7. ^{13}C NMR (75 MHz, DMSO-d_6) spectrum of the compound (**3**).

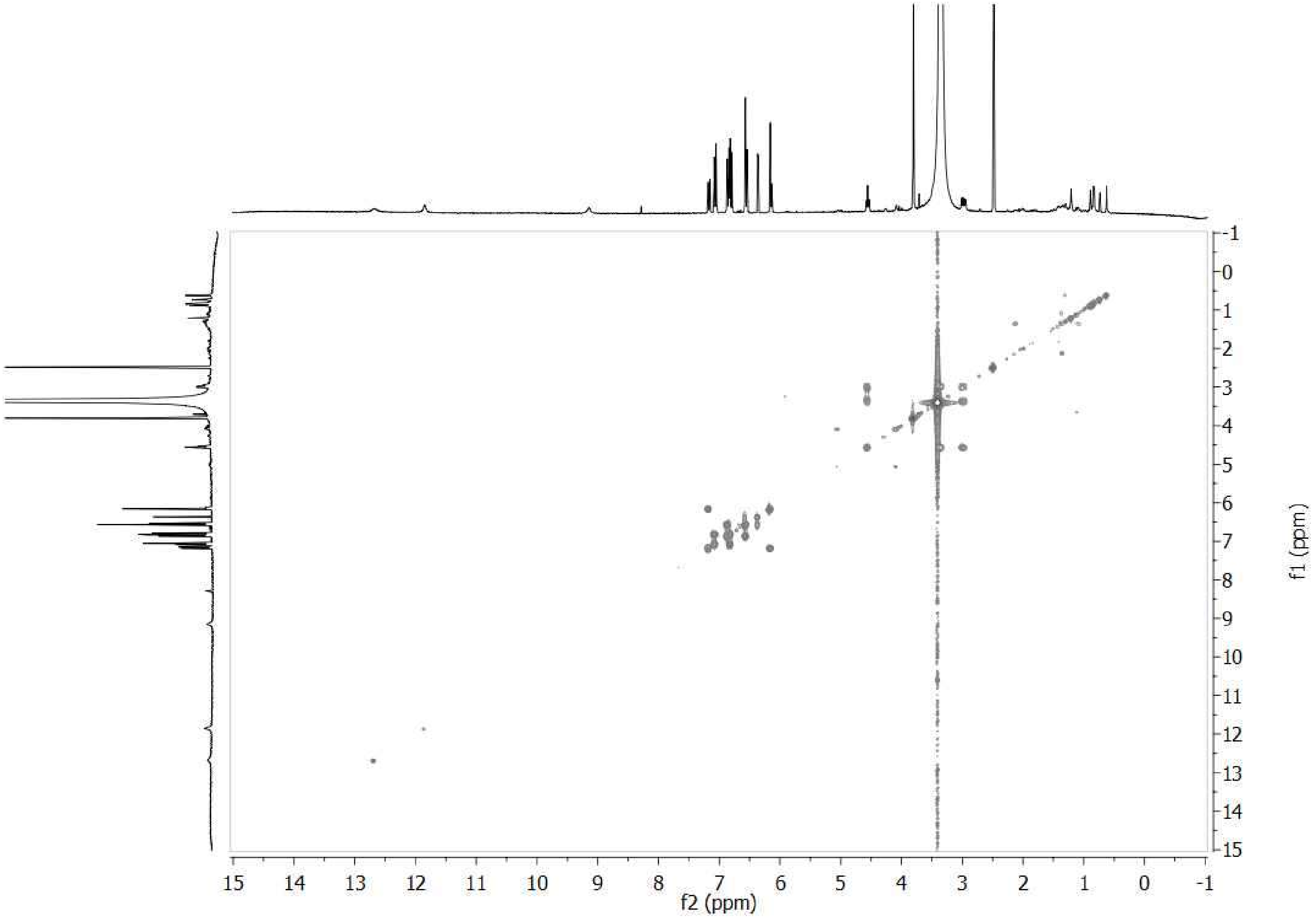

Figure S8. COSY (DMSO-d_6) spectrum of the compound (**3**).

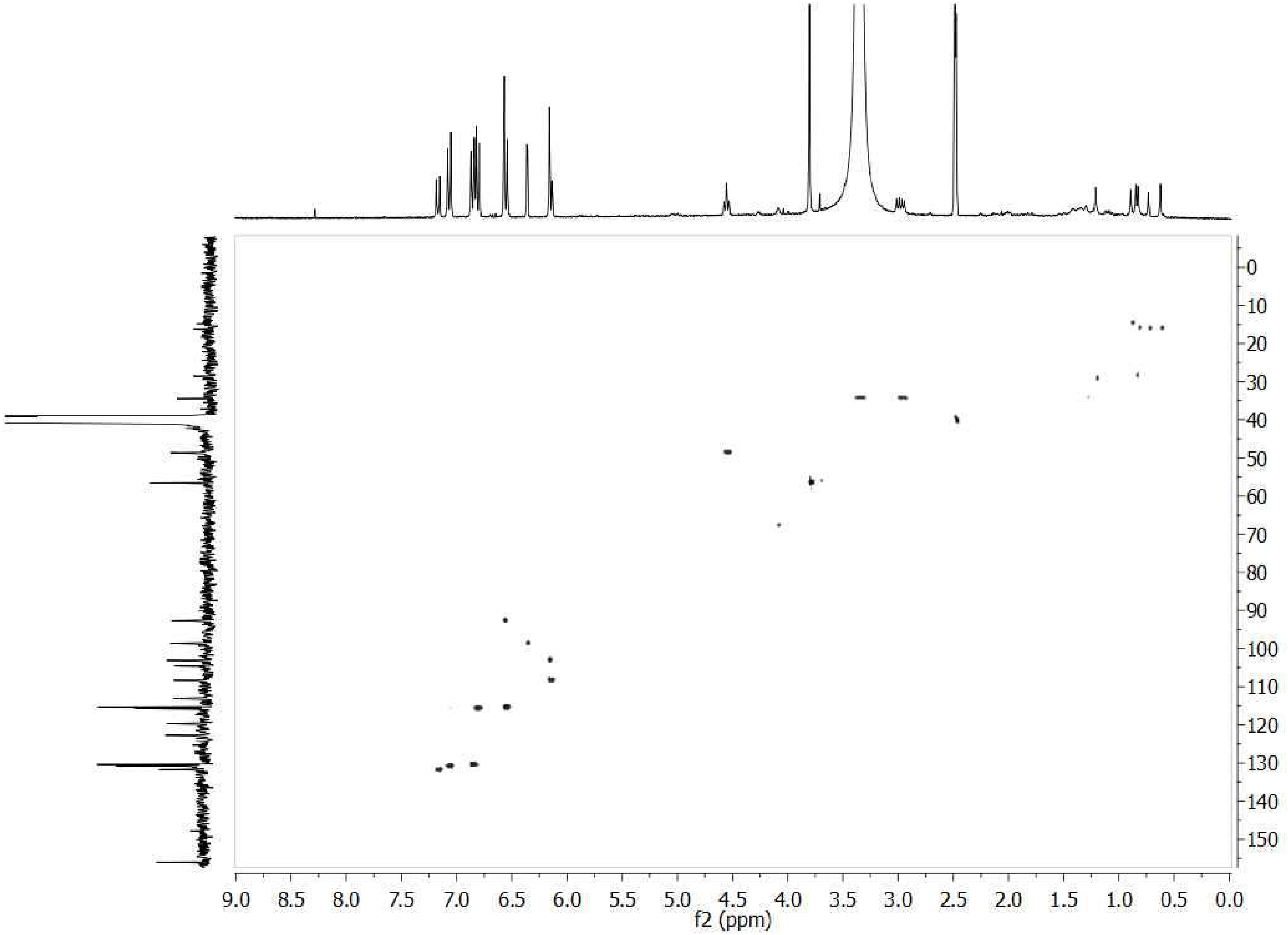

Figure S9. HSQC (DMSO-d_6) spectrum of the compound (**3**).

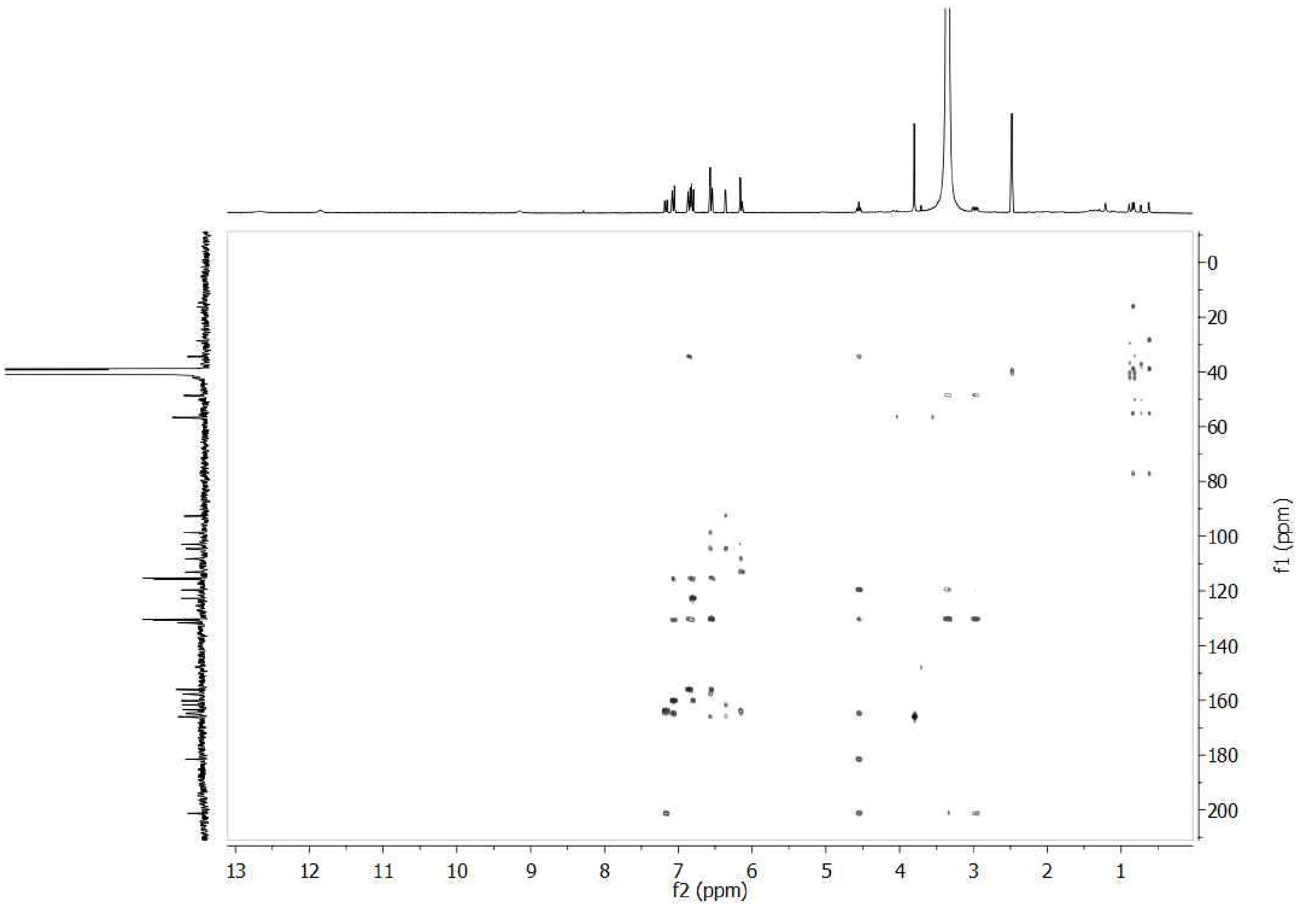

Figure S10. HMBC (DMSO-d_6) spectrum of the compound (**3**).

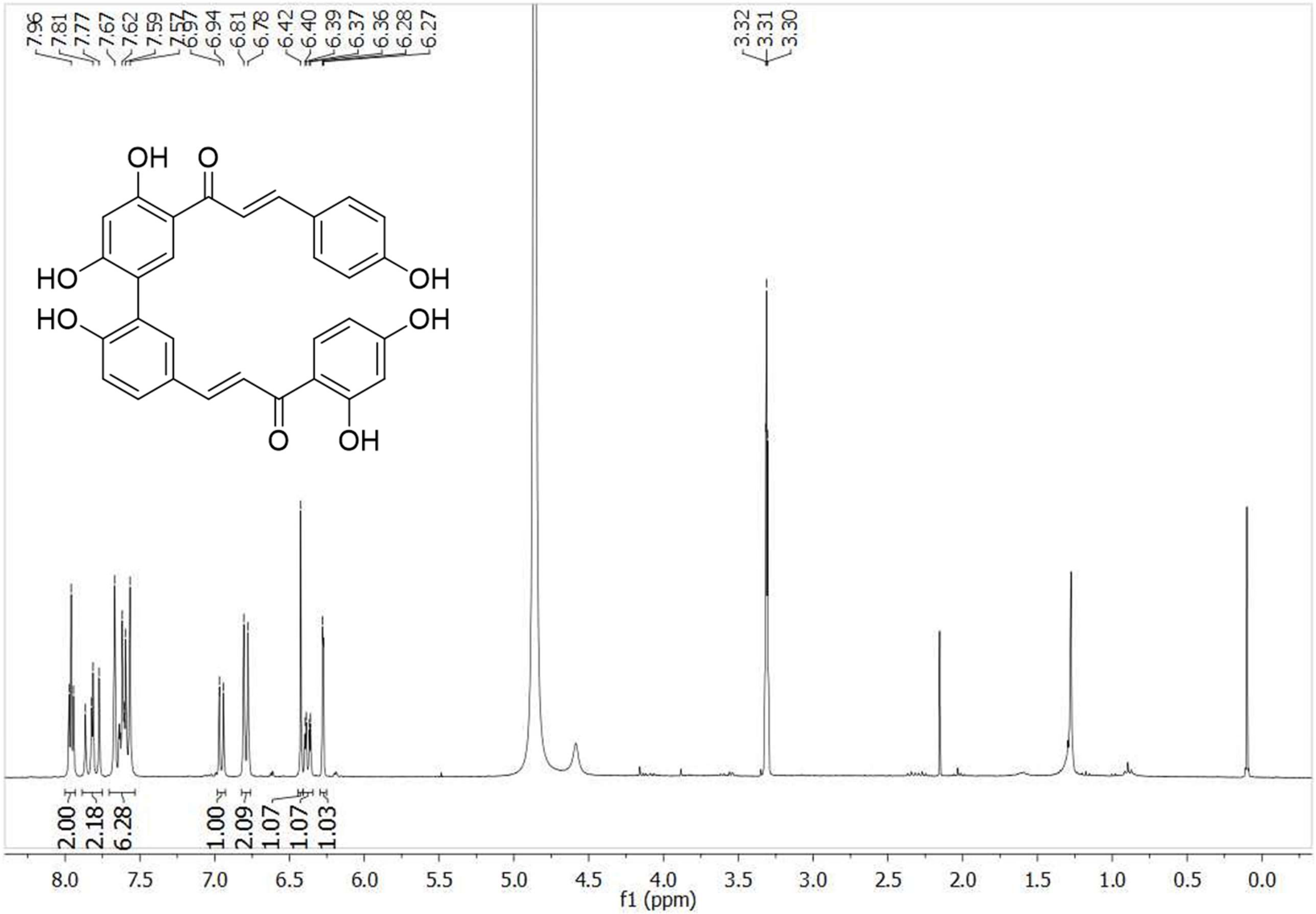

Figure S11. ^{1}H NMR (300 MHz, CD_3OD) spectrum of the compound (**4**).

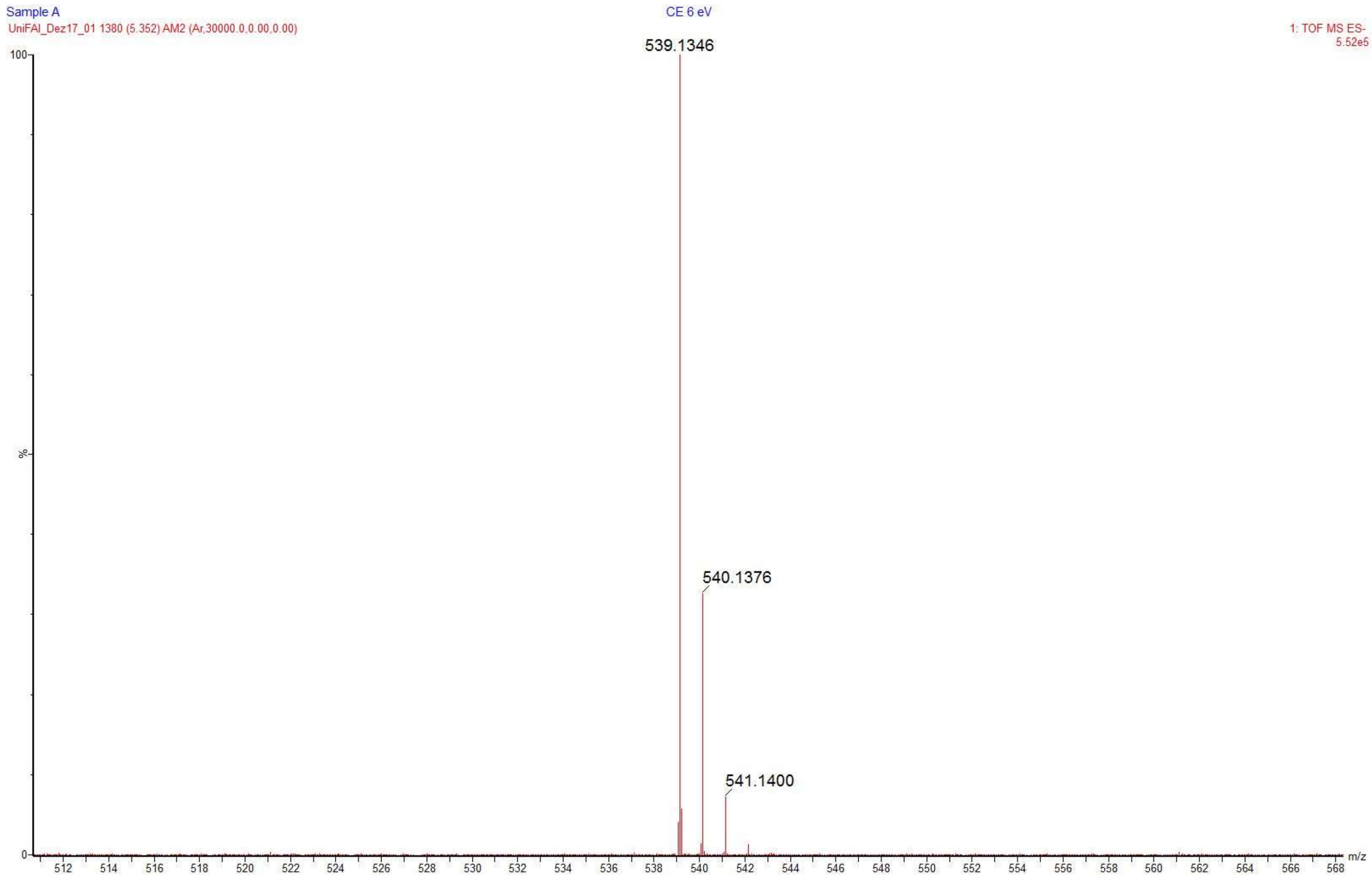

Figure S12. Mass spectrum of the compound (**2**).

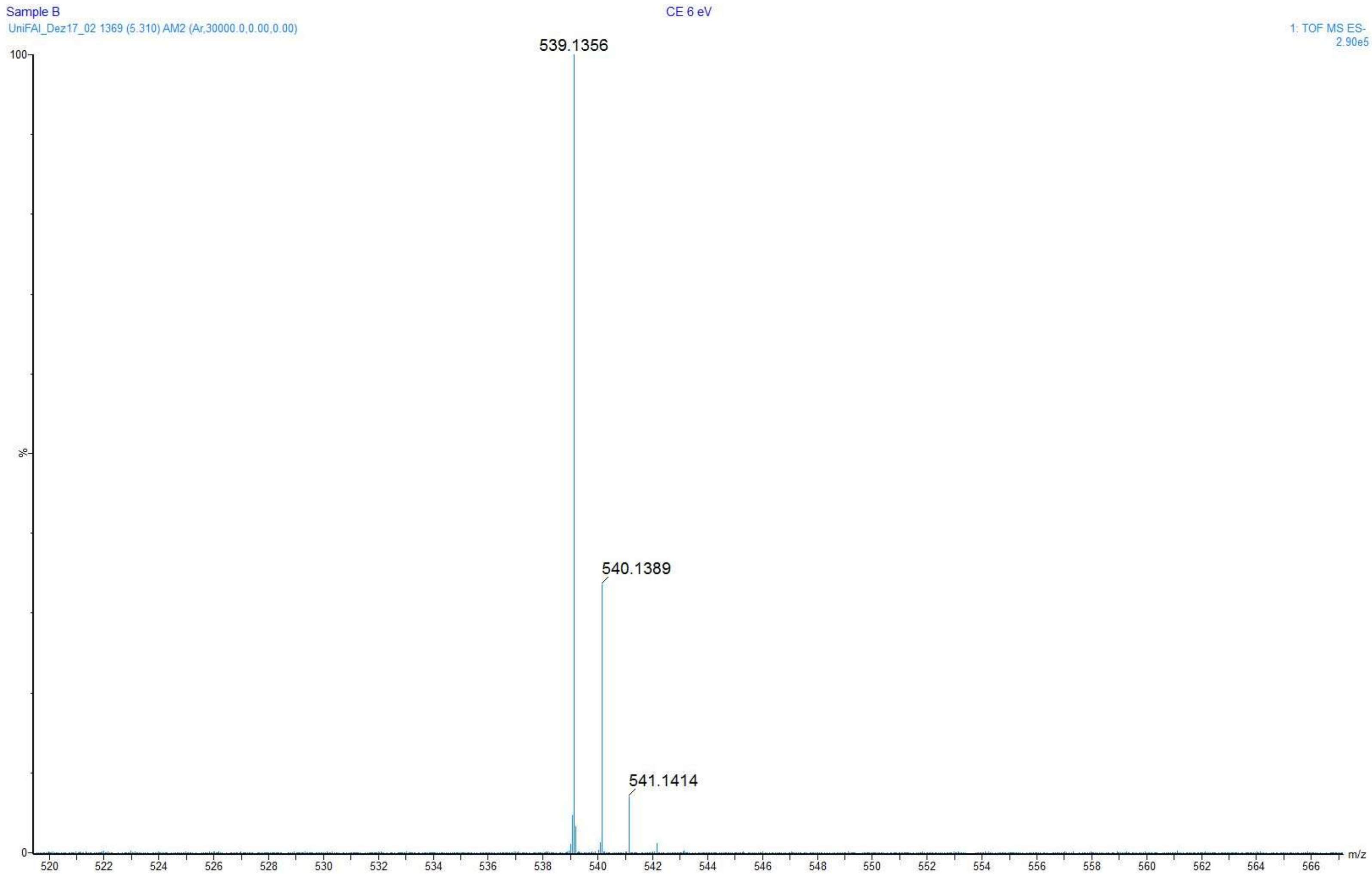

Figure S13. Mass spectrum of the compound (**3**).

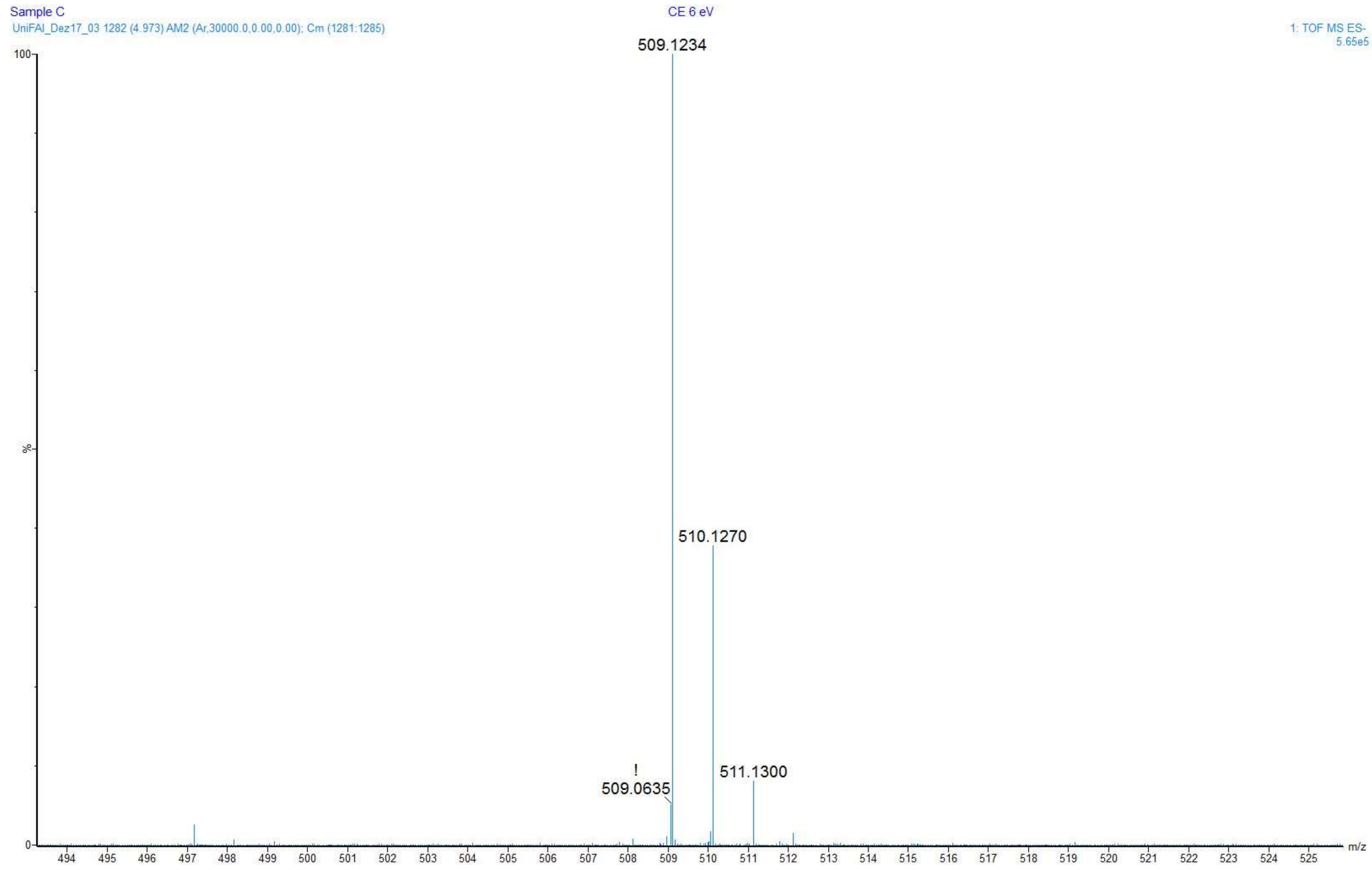

Figure S14. Mass spectrum of the compound (**4**).

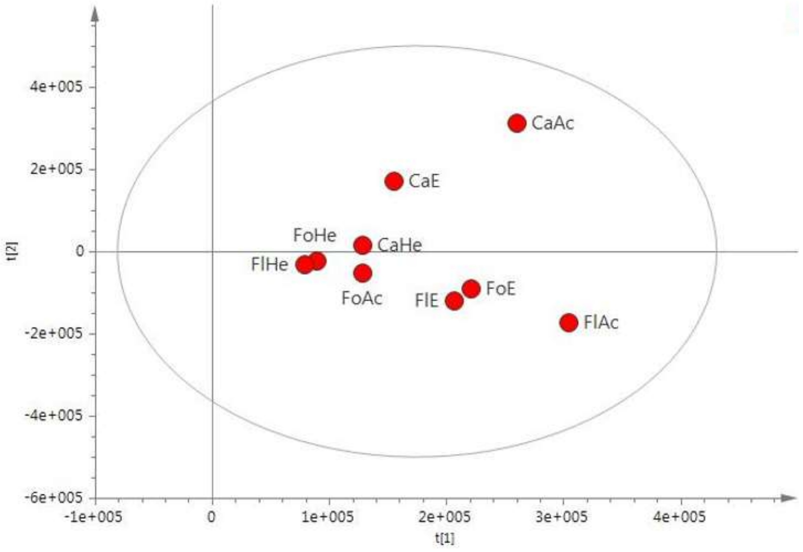

Figure S15. Principal component analysis (PCA) of samples from *P. pluviosa.*
